# Characterization of a Novel Nicotine Degradation Gene Cluster *ndp* in *Sphingomonas melonis* TY and Its Evolutionary Analysis

**DOI:** 10.3389/fmicb.2017.00337

**Published:** 2017-03-09

**Authors:** Haixia Wang, Xiao-Yang Zhi, Jiguo Qiu, Longxiang Shi, Zhenmei Lu

**Affiliations:** ^1^Institute of Microbiology, College of Life Sciences, Zhejiang UniversityHangzhou, China; ^2^Yunnan Institute of Microbiology, School of Life Sciences, Yunnan UniversityKunming, China; ^3^Department of Microbiology, College of Life Sciences, Nanjing Agricultural UniversityNanjing, China; ^4^Institution of System Engineering, College of Computer Science and Technology, Zhejiang UniversityHangzhou, China

**Keywords:** nicotine, *Sphingomonas melonis* TY, *ndp* gene cluster, pathway evolution, pathway distribution

## Abstract

*Sphingomonas melonis* TY utilizes nicotine as a sole source of carbon, nitrogen, and energy through a variant of the pyridine and pyrrolidine pathways (VPP). A 31-kb novel nicotine-degrading gene cluster, *ndp*, in strain TY exhibited a different genetic organization with the *vpp* cluster in strains *Ochrobactrum rhizosphaerae* SJY1 and *Agrobacterium tumefaciens* S33. Genes in *vpp* were separated by a 20-kb interval sequence, while genes in *ndp* were localized together. Half of the homolog genes were in different locus in *ndp* and *vpp*. Moreover, there was a gene encoding putative transporter of nicotine or other critical metabolite in *ndp*. Among the putative nicotine-degrading related genes, the nicotine hydroxylase, 6-hydroxy-L-nicotine oxidase, 6-hydroxypseudooxynicotine oxidase, and 6-hydroxy-3-succinyl-pyridine monooxygenase responsible for catalyzing the transformation of nicotine to 2, 5-dihydropyridine in the initial four steps of the VPP were characterized. Hydroxylation at C6 of the pyridine ring and dehydrogenation at the C2–C3 bond of the pyrrolidine ring were the key common reactions in the VPP, pyrrolidine and pyridine pathways. Besides, VPP and pyrrolidine pathway shared the same latter part of metabolic pathway. After analysis of metabolic genes in the pyridine, pyrrolidine, and VPP pathways, we found that both the evolutionary features and metabolic mechanisms of the VPP were more similar to the pyrrolidine pathway. The linked *ndpHFEG* genes shared by the VPP and pyrrolidine pathways indicated that these two pathways might share the same origin, but variants were observed in some bacteria. And we speculated that the pyridine pathway was distributed in Gram-positive bacteria and the VPP and pyrrolidine pathways were distributed in Gram-negative bacteria by using comprehensive homologs searching and phylogenetic tree construction.

## Introduction

Nicotine is the most abundant alkaloid in tobacco plants, and it maintains a high content (15.680–32.536 mg/g dry weight according to the particle size of the solid powdery waste) in tobacco waste, which accumulates in large amounts (Civilini et al., 1997; Novotny and Zhao, 1999; Cosic et al., 2012). Nicotine has good hydrophilicity, can spread in the environment through water and soil, and poses a threat to human health and the environment because of its toxicity. Moreover, nicotine can be easily absorbed and traverse the blood-brain barrier (Oldendorf, 1974; Oldendorf et al., 1979; O'neill et al., 2002; Lemay et al., 2004), and it has been classified as a Toxics Release Inventory (TRI) chemical by the United States Environmental Protection Agency since 1994. Microbial bioremediation has been considered an economical and effective way to eliminate nicotine in the environment (Brandsch, 2006; Gurusamy and Natarajan, 2013).

To date, four main types of nicotine degradation pathways have been described in microorganisms. First, the pyrrolidine pathway (PRL) was found in *Pseudomonas* sp. No. 41, *Pseudomonas putida* NRRL B-8061, *Pseudomonas putida* S16, *Pseudomonas* sp. HF-1, and *Pseudomonas* sp. HZN6 (Wada and Yamasaki, 1953; DeTraglia and Tometsko, 1980; Wang et al., 2004; Ruan et al., 2005; Qiu et al., 2011). Second, the pyridine pathway (PD) was reported in *Arthrobacter oxydans* P-34, *Arthrobacter nicotinovorans* pAO1, *Nocardioides* sp. JS614, *Rhodococcus opacus* B4, and *Arthrobacter aurescens* M2012083 (Hochstein and Rittenberg, 1959a; Ganas et al., 2008; Cobzaru et al., 2011; Yao et al., 2012, 2015). Third, the methyl pathway was found only in several fungi *Microsporum gypseum, Pellicularia filamentosa* JTS-208, *Cunninghamella echinulata* IFO-4444, and *Aspergillus oryzae* 112822 (Sindelar et al., 1979; Uchida et al., 1983; Meng et al., 2010). Fourth, a variant of the pyridine and pyrrolidine pathway (VPP) was discovered in *Agrobacterium tumefaciens* S33, *Sphingomonas melonis* TY, *Shinella* sp. HZN7, and *Ochrobactrum rhizosphaerae* SJY1 (Wang et al., 2009, 2011; Ma et al., 2013; Yu et al., 2014). Additionally, two special pathways were recently found in *Pseudomonas plecoglossicida* TND35 and *Pusillimonas* sp. T2, the former with some new intermediates and the latter containing both the VPP and a partial PD with the formation of 2, 6-dihydroxypyridine (Raman et al., 2013; Ma et al., 2014). Interestingly, four intermediates of the nicotine metabolic pathway defined as 6-hydroxynicotine (6HN), pseudooxynicotine, 3-succinoyl-pyridine, and 6-hydroxy-3-succinoyl-pyridine (HSP) were studied in a nicotine-degrading strain *Achromobacter nicotinophagum* in 1958 (Hylin, 1958, 1959). It was anticipated that more new pathways would be found. Among these published pathways, the most well-established were the PD in *A. nicotinovorans* pAO1 (Dang et al., 1968; Brühmüller et al., 1972; Grether-Beck et al., 1994; Schenk et al., 1998; Baitsch et al., 2001; Chiribau et al., 2004, 2006; Sachelaru et al., 2005, 2006; Mihasan et al., 2007), the PRL in *Pseudomonas putida* S16 (Tang et al., 2008, 2009, 2011, 2012, 2013; Jiang et al., 2015), and the VPP in *O. rhizosphaerae* SJY1 (Yu et al., 2014, 2015). The nicotine-degrading gene clusters, for example, the *nic*-genes in *A. nicotinovorans* pAO1, *nic1* and *nic2* in strain S16, and the *vpp* cluster in strain SJY1, have been comprehensively studied.

The nomenclature and classification of microbial nicotine degradation pathway was according to the reaction position of the first step in each pathway. In the PRL, the first reaction was occurred at C2–C3 bond of the pyrrolidine ring (Tang et al., 2013); in the PD, the first reaction was occurred at C6 of the pyridine ring (Hochstein and Rittenberg, 1959b); in the methyl pathway, the first reaction was occurred at methyl group linked in the N atom of the pyrrolidine ring (Uchida et al., 1983). All these three pathways have totally different nicotine metabolism pathway from the first step of degradation (Raman et al., 2013). And VPP was designated by its feature of combing upper pathway of PD with lower pathway of PRL (Wang et al., 2012). There are two key steps shared by the PRL, PD, and VPP pathways, that were hydroxylation at C6 of the pyridine ring and dehydrogenation at the C2–C3 bond of the pyrrolidine ring, which was important for opening the ring.

Horizontal gene transfer (HGT) has been recognized as a main force in the genomes evolution for a long time (Gray, 1992). In comparison to eukaryotes, which evolve mainly through the modification of existing genetic information, bacteria obtain a considerable ratio of their genetic variants through HGT from distantly related organisms (Ochman et al., 2000). There are some commonly used criteria and methods for identifying HGT. HGT creates an unduly high degree of DNA or protein sequence similarity between the donor and the recipient strains for the character in question, and the acquired trait will be limited to the offsprings of the recipient strain and absent from the closely related taxa, thereby producing a scattered phylogenetic distribution (Ochman et al., 2000). However, the strongest evidence to identify cases of HGT derives from a molecular genetic analysis of their DNA sequences. Bacterial species display a wide range of variation in their total G+C content, but the genes in a particular species' genome are considerably similar in regard to their nucleotide compositions, patterns of codon usage and frequencies of di- and tri-nucleotides (Sueoka, 1962; Muto and Osawa, 1987; Karlin et al., 1998). Therefore, sequences that are newly transferred into the bacterial genome, namely, those introduced through HGT, retain the sequence characteristics of the original genome and have atypical nucleotide compositions, or patterns of codon usage bias with the host genome and thus can be differentiate from vertically inherited DNA (Lawrence and Ochman, 1998). Additionally, the regions contiguous to the genes that were confirmed to be horizontally transferred often contained traces of sequences promoting their integration, such as mobile element remnants, transfer origins of plasmids or attachment sites of phage integrases, further confirming their foreign origin in the genome (Ochman et al., 2000).

Although the molecular mechanism of nicotine metabolism is very clear in several representative strains, the evolutionary relationship of these nicotine degradation gene clusters and the evolutionary relationship between the PD, PRL, and VPP remain unclear. More gene clusters must be discovered to help unravel the evolutionary relationships. Moreover, some intermediates that form during nicotine metabolism have pharmacological value (Roduit et al., 1997; Wang et al., 2005; Goetz and Garg, 2013), necessitating additional genetic and metabolic resources for industrial applications. In this work, we studied a novel nicotine-degrading gene cluster, *ndp*, in *S. melonis* TY. This 31-kb *ndp* in strain TY exhibited a different genetic organization with the *vpp* cluster in strains SJY1 and S33. Genes in *vpp* were separated by a 20-kb interval sequence, while genes in *ndp* were localized together. Half of the homolog genes were in different locus in *ndp* and *vpp*. Moreover, there was a gene encoding putative transporter of nicotine or other critical metabolite in *ndp*, while missing in *vpp*. The amino acid sequence identity between two key common enzymes was low, 45 and 54% to NdpA_*L*_ and NdpB, respectively. All these differences showed that *ndp* was special and had a different evolution process with that of *vpp*. Nicotine dehydrogenase *ndpA*, 6-hydroxynicotine oxidase *ndpB*, 6-hydroxypseudooxynicotine oxidase *ndpC* and 6-hydroxy-3-succinoyl-pyridine 3-monooxygenase *ndpD* were identified. In a word, *ndp* was found to be an integrated and compact nicotine-degrading gene cluster with a different genetic organization and coding sequence compared with *vpp* in strains SJY1 and S33. We analyzed the three nicotine-degrading gene clusters in the VPP and found that all of them seemed to evolve via HGT. Analysis of the origin of the three main pathways involved in nicotine degradation revealed that the VPP was more similar to PRL.

## Materials and methods

### Chemicals and reagents

(*S*)-Nicotine (>99%) was obtained from Chemsky international Co., Ltd (Shanghai, China). 6HN, 6-hydroxypseudooxynicotine (6HPON), and HSP were prepared as previously described (Ma et al., 2013). TransStart® FastPfu DNA Polymerase for fragment amplification was purchased from TransGen Biotech (Beijing, China). Restriction enzymes used for plasmid construction and premixed protein marker for protein electrophoresis were purchased from Takara Biotechnology Co., Ltd. (Dalian, China). Antibiotics, isopropyl β-D-1-thiogalactopyranoside (IPTG) and other reagents were purchased from Shanghai Sangon Biotech Co., Ltd. (Shanghai, China). The plasmid extraction, gel extraction, and DNA purification kits were obtained from Omega Bio-tek, Inc. (Norcross, GA, USA). Bacterial genomic DNA was extracted using the TIANamp Bacteria DNA Kit from Tiangen Biotech co., Ltd. (Beijing, China). All reagents and solvents were of analytical or chromatographic grade.

### Bacterial strains, plasmids, and growth conditions

The bacterial strains and plasmids used in this study are listed in Table S1 and the primers are shown in Table S2. The wild-type strain *S. melonis* TY (deposited in China General Microbiological Culture Collection Center, collection number CGMCC1.15791) can use nicotine as a sole carbon, nitrogen, and energy source to grow (Wang et al., 2011). The wild-type strain TY and its derivatives were cultured aerobically in LB medium or inorganic salt medium (ISM) supplemented with nicotine at 30°C as described previously (Wang et al., 2011). *Escherichia coli* strains were grown in LB broth at 37°C. When necessary, ampicillin, kanamycin, and tetracycline were used at final concentrations of 100, 50, and 10 μg/mL, respectively, excluding Origami B(DE3) (tetracycline and kanamycin were used at final concentrations of 12.5 and 15 μg/mL, respectively). IPTG was used as an inducer at a given concentration. The 2, 6-diaminopimelic acid (2, 6-DAP) was used at a final concentration of 0.3 mM for *E. coli* WM3064. Competent *E. coli* WM3064 were prepared according to standard methods using 0.1 M CaCl_2_ in 20% (v/v) glycerol (Ausbel et al., 1995).

### Genome sequence analysis and prediction of nicotine metabolism-related genes in strain TY

To identify putative genes involved in nicotine degradation in strain TY, we conducted a BLAST analysis against the draft genome sequence of strain *S. melonis* TY (the report about the draft genome sequence of strain TY is under reviewed in Frontiers in Microbiology, and the accession number of the genome is LQCK00000000) using known nicotine metabolic genes, such as *ndhLSM* and *6hlno* in *A. nicotinovorans* (Grether-Beck et al., 1994) and *hspA, hspB, hpo, nfo, ami*, and *iso* in *Pseudomonas putida* S16 (Tang et al., 2008, 2011, 2012). The hits were designated as putative nicotine metabolism-related genes in strain TY and used for reverse transcription quantitative PCR (RT-qPCR) analysis. Subsequently, some of them were chosen for gene disruption experiments.

### RT-qPCR analysis of *ndpA, ndpB, ndpC*, and *ndpD*

ISM with 1 g/L glucose and 1 g/L (NH_4_)_2_SO_4_ was used as the control medium for the RT-qPCR experiments. ISM supplemented with 1 g/L nicotine was established as the experimental group. Cultures in the presence and absence of nicotine were cultured in triplicate at 30°C to mid-exponential phase. Total RNA were extracted using the RNAprep Pure Bacteria Kit (Tiangen Biotech, Beijing, China) and reverse-transcribed into cDNA using the random hexamer primers and PrimeScript RT Reagent Kit with gDNA Eraser (Perfect Real Time; Takara, Dalian, China). The respective cDNA fragments were applied as templates in the PCR using the gene-specific primers shown in Table S2 designed using Beacon Designer 7 software, Premier Biosoft International (Palo Alto, CA, USA). qPCR was performed on a Rotor-Gene Q real-time PCR detection system (Qiagen, Germany) using TransStart Top Green qPCR SuperMix (TransGen Biotech, Beijing, China). The genome of strain TY was used as a positive control, and untranscribed RNA was used as a negative control. Melting curves and agarose gel analyses were used to confirm the specificity of the PCR products. The threshold cycle (C_T_) values for each target gene were normalized to the 16S rRNA reference gene. The 2^ΔΔCT^ method was used to calculate the relative expression level, where ΔΔC_T_ = (C_T, target_ − C_T, 16S_)_induction_ − (C_T, target_ − C_T, 16S_)_control_, whereas the theoretical efficiency value 2 was replaced with the estimated PCR efficiency value (Livak and Schmittgen, 2001; Ramakers et al., 2003; Ruijter et al., 2009; Tuomi et al., 2010). In brief, the PCR data were saved in “LinReg Export Format” in the Rotor-Gene Q series software program and imported into the LinRegPCR program. Individual samples were then checked sequentially, and the mean PCR efficiency values were used for each amplicon group as a substitute for the theoretical efficiency value 2.

### Gene knockout and complementation

All the vectors constructed in this work were first simulated using Vector NTI Advance 11.5.1 (Invitrogen, USA). Specific primers for fragment amplification were annotated in this software and used as output for synthesis, as were the primers used to sequence the constructed vectors. The sequencing results obtained from the biotechnology company were assembled and checked for mutations with simulative construction by Vector NTI Advance 11.5.1. In-frame disruption of *ndpA*_*L*_, *ndpB, ndpC*, and *ndpD* in strain *S. melonis* TY was performed using the suicide plasmid pEX18Tc and a two-step homologous recombination method (Chen et al., 2014). Plasmids pEX18Tc-*ndpA*_*L*_, pEX18Tc-*ndpB*, pEX18Tc-*ndpC*, and pEX18Tc-*ndpD* for gene knockout were constructed by fusing the PCR products of the kanamycin resistance gene and two upstream and downstream fragments of the target gene, amplified with the primers shown in Table S2, to *Sac* I and *Hin*d III-digested pEX18Tc using the In-Fusion® HD Cloning Kit (Takara, Dalian, China). Constructed plasmids were transformed into *E. coli* DH5α and sequenced, and accurate constructions were preserved and used for subsequent analyses. These plasmids were then transformed into *E. coli* WM3064 (2, 6-DAP auxotroph; Dehio and Meyer, 1997; Saltikov and Newman, 2003) before being conjugated to strain TY as described previously (Saltikov and Newman, 2003). The TYΔ*ndpA*_*L*_, TYΔ*ndpB*, TYΔ*ndpC*, and TYΔ*ndpD* recombinants were screened on LB plates containing kanamycin and then verified using specific primers for PCR and sequencing. If double-crossover recombinants were not acquired during the first cycle of screening, the single crossover recombinants were cultured in liquid LB containing kanamycin and 10% sucrose (*w*/*v*) for several generations and then screened on LB plates supplemented with kanamycin and 10% sucrose (*w*/*v*) to obtain single colonies. Verification was performed as mentioned above until double-crossover recombinants were obtained.

Plasmids pRK415-*ndpA*_*L*_, pRK415-*ndpB*, pRK415-*ndpC*, and pRK415-*ndpD* for gene complementation were constructed by fusing the PCR products corresponding to the full-length *ndpA*_*L*_, *ndpB, ndpC*, and *ndpD* amplified with the primers shown in Table S2, to *Hin*d III and *Eco*R I-digested pRK415. After sequencing and obtaining the desired construction, four plasmid constructs were transformed into *E. coli* WM3064 and then mated into the TYΔ*ndpA*_*L*_, TYΔ*ndpB*, TYΔ*ndpC*, and TYΔ*ndpD* strain by conjugation to obtain TYΔ*ndpA*_*L*_(pRK415-*ndpA*_*L*_), TYΔ*ndpB*(pRK415-*ndpB*), TYΔ*ndpC*(pRK415-*ndpC*), and TYΔ*ndpD*(pRK415-*ndpD*), respectively.

### Cell growth and resting cell reactions of TY and its derivatives

The four mutants TYΔ*ndpA*_*L*_, TYΔ*ndpB*, TYΔ*ndpC*, and TYΔ*ndpD* were examined for their ability to grow in the presence of nicotine. After gene complementation, all the complementary strains were inoculated in ISM medium supplemented with nicotine to determine whether they restored nicotine-degrading ability. After preliminarily confirming the importance of these four genes in nicotine degradation of strain TY, biotransformation tests were conducted using TYΔ*ndpA*_*L*_, TYΔ*ndpB*, TYΔ*ndpC*, TYΔ*ndpD*, wild type TY, and an inactivated strain of wild type TY (heated at 80°C for 5 min to exclude nicotine absorption; Harwood et al., 1994) and a control group containing only nicotine, to determine the intermediate product of nicotine produced by these four mutant strains. The cells were harvested by centrifugation at 6,000 × g for 5 min and washed twice with 12 mM phosphate-buffered saline (PBS, pH 7.4). Subsequently, the cells pellets were resuspended in ISM (resting cells). The biotransformation test was performed in a 150-mL beaker flask containing 60 mg dry cell weight of resting cells (with an optical density at 600 nm (OD_600nm_) of 5.0, in which one OD_600nm_ unit was equivalent to 0.40 g/L dry cell weight) and 0.5 mg/mL nicotine in 30 mL of sterilized ISM and incubated at 30°C and 200 rpm for 2 days. The reactions were stopped by centrifugation at 6,000 × g for 5 min, and the supernatant was subjected to spectrum scanning and liquid chromatography-mass spectrometry (LC-MS) analysis. Biotransformation tests were conducted as described for TYΔ*ndpA*_*L*_, TYΔ*ndpB*, TYΔ*ndpC*, and TYΔ*ndpD* with 6HN, 6HPON, and HSP. Additionally, growth ability was examined using these four mutants with nicotine-degrading intermediates (6HN, 6HPON, and HSP) in strain TY.

### Heterologous expression of *ndpA, ndpB, ndpC*, and *ndpD*

To verify the function of *ndpA*, heterologous expression of NdpA was performed according to the method described for the heterologous expression of *vppA* (Yu et al., 2015). *P. putida* KT2440 and *Sphingomonas aquatilis* JSS7^T^ were chosen as the expression host (details concerning the heterologous expression of *ndpA, ndpB*, and *ndpD* are provided in the supporting information).

pET-28a(+) and pET-22b(+) were used as the expression vectors for *ndpB*, and *E. coli* BL21(DE3) and Origami B(DE3) were used as the expression hosts. Origami B(DE3) was selected because it carries glutathione reductase (*gor*) and thioredoxin reductase (*trxB*) mutations to enhance the formation of disulfide bonds in the *E. coli* cytoplasm (Prinz et al., 1997; Aslund et al., 1999).

For heterologous expression of NdpB, pRK415-*ndpB* was mated into *P. putida* KT2440 and *Sphingomonas aquatilis* JSS7^T^ through *E. coli* WM3064 to generate *P. putida* KT-*ndpB* and *Sphingomonas*-*ndpB*, respectively. The method applied for whole cell transformation of 6HN by NdpB was the same as that described for NdpA. For homologous expression of NdpB, His_6_-tagged fusion protein under the promoter of pRK415 was expressed in strain TYΔ*ndpB* and purified by Wuhan GeneCreate Biological Engineering Co., Ltd. (Wuhan, China) under native conditions. The obtained protein was evaluated by western blot analysis and detection of activity.

Heterologous expression of NdpC was conducted as described for NdpA and NdpB, with the transfer of pRK415-*ndpC* into *P. putida* KT2440 and *Sphingomonas aquatilis* JSS7^T^ to obtain *P. putida* KT-*ndpC* and Sphingomonas-*ndpC*; the substrate was substituted with 6HPON when performing whole cell transformation to detect the enzyme activity.

C-terminal and N-terminal His_6_-tagged expressions of NdpD were performed by fusing the *ndpD* (full-length gene or without the stop codon) product to *Nco* I and *Hin*d III-digested pET-28a(+) or *Nde* I and *Xho* I-digested pET-22b(+) to obtained pET28a-*ndpD*-C and pET22b-*ndpD*-N, respectively, using *E. coli* BL21(DE3) as the expression host. The crude enzyme activity of NdpD was detected according to Yu et al. (2014).

NdpD was also heterologously expressed in *P. putida* KT2440 and *Sphingomonas aquatilis* JSS7^T^ with pRK415-*ndpD* as described above. Whole cell transformation of HSP by *P. putida* KT-*ndpD* and *Sphingomonas*-*ndpD* was also conducted as described for NdpA.

### Analytical methods

Biotransformation of nicotine by resting cells was analyzed by spectrum scanning using a Lambda25 UV/VIS spectrometer (PerkinElmer, USA). LC-MS analysis was performed using liquid chromatography (Agilent 1200, USA) equipped with Sielc Obelisc_N column (5 μm, 2.1 × 150 mm) and a LCQ Deca XP Max MS instrument (Thermo Finigan) with an electrospray interface (Turbo Ion Spray). The iron spray voltage was set at 4,500 V. Nitrogen was used as the sheath gas (55 arb) and auxiliary gas (5 arb). The capillary temperature was set at 275°C, and the capillary voltage was set at 41 V. Mobile phase A was 50 mM ammonium acetate, pH 5.0, adjusted with formic acid, and mobile phase B was acetonitrile. The system was run as follows: 0–5 min, 5% A+95% B; 5–35 min, from 5% A+95% B to 70% A+30% B; 35–45 min, 70% A+30% B. The total flow rate was 0.3 mL/min, and 15 μl of the sample was injected. The column was set at 35°C, and the detection was performed at 254 nm. The obtained data were analyzed using Xcalibur software (Thermo Electron Corporation). The HPLC analysis was performed according to Ruan et al. (2005).

### Genetic characteristics of genes in the VPP

The G+C content of genome of TY, SJY1, and S33 were searched at NCBI (http://www.ncbi.nlm.nih.gov/), and the G+C content of the nicotine-degrading gene cluster was calculated at DNA/RNA GC Content Calculator in EndMemo (http://www.endmemo.com/bio/gc.php). CAIcal (Puigbò et al., 2008) and condonW (http://codonw.sourceforge.net/index.html) were used to calculate the nucleotide composition and relative synonymous codon usage (RSCU) of the nicotine-degrading genes, and condonW was also used to calculated the effective number of codons (ENC) value. BLASTp was used to search for closely related taxa of the nicotine-degrading gene of strain TY, SJY1, and S33 (https://blast.ncbi.nlm.nih.gov/Blast.cgi). HGT remnants were checked by searching the annotation of contiguous genes of nicotine-degrading gene clusters.

### Analysis of evolutionary relationships among nicotine degradation pathways

Hydroxylation at C6 of the pyridine ring and dehydrogenation at the C2–C3 bond of the pyrrolidine ring were common reactions in the VPP, PRL, and PD pathways. Therefore, all members of the corresponding hydroxylases and dehydrogenases identified in previous experiments were used as queries in BLAST searches against a local genomes database that included 2806 predicted prokaryotic proteomes (downloaded from the NCBI FTP server, ftp://ftp.ncbi.nih.gov/). The genomes with homologs of both NdpA_*L*_ and NdpB were selected, and the corresponding protein sequences were retrieved for subsequent analyses. The NdpHFEG proteins were also determined using the BLAST method. The protein sequences of NdpA_*L*_ and NdpB were aligned using ClustalW (Larkin et al., 2007), and the resulting alignments of individual proteins were used to infer the organismal phylogeny with the maximum likelihood algorithm (ML) in the MPI-parallelized version of RAxML version 7.3.0 (Stamatakis, 2006). Ambiguous alignments were removed using the Gblocks method in SEAVIEW (Gouy et al., 2010) with options for a less stringent selection. The LG model (Le and Gascuel, 2008) with a proportion of invariable sites (+I), a gamma-shaped distribution of rates across sites (+G) and observed amino acid frequencies (+F) was used for the phylogeny inference. The topologies of the phylogenetic trees were evaluated using the bootstrap resampling method of Felsenstein (Felsenstein, 1985) with 100 replicates.

### Accession number of nucleotide sequence

The sequence of the *ndp* cluster from strain TY is available in GenBank under accession number LQCK02000019.1.

## Results

### A putative nicotine metabolism gene cluster is present in the genome of strain TY

**Figure 1:**
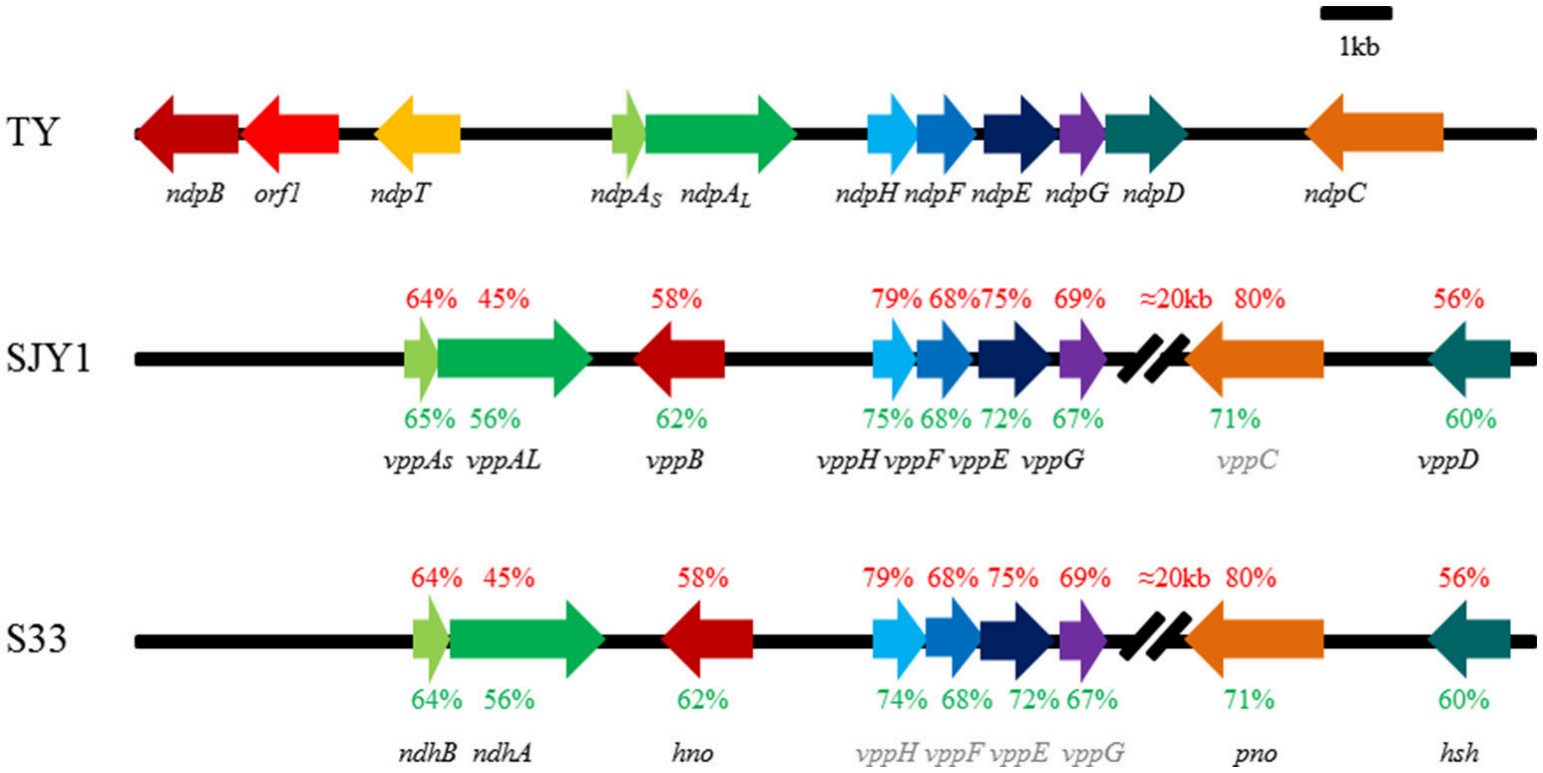
**Genetic organization and amino acid sequence identity of nicotine degradation gene clusters in ***Sphingomonas melonis*** TY, ***Ochrobactrum rhizosphaerae*** SJY1, and ***Agrobacterium tumefaciens*** S33**. The arrows indicate the size and direction of the transcription of each gene, and genes with the same fill color are isoenzymes. The gene name in gray represents speculated genes in the corresponding strain. The corresponding amino acids sequence identity between TY and S33, TY and SJY1 are shown in red text, and the nucleotide sequence identity is shown in green. *ndpAs*, nicotine hydroxylase, subunit S; *ndpA*_*L*_, nicotine hydroxylase, subunit L; *ndpB*, 6-hydroxy-L-nicotine oxidase; *ndpC*, 6-hydroxypseudooxynicotine oxidase; *ndpD*, HSP monooxygenase; *ndpE*, 2,5-DHP dioxygenase; *ndpF, N*-formylmaleamic acid deformylase; *ndpG*, maleamate amidase; *ndpH*, maleate isomerase; Percent amino acid sequences identity of genes compared with orthologous gene product from TY were labeled above the gene cluster in SJY1 and S33, respectively. TY, *Sphingomonas melonis* TY; SJY1, *Ochrobactrum rhizosphaerae* SJY1; and S33, *Agrobacterium tumefaciens* S33.

After performing a BLAST analysis against the genome of strain TY using previously known nicotine metabolism genes, we found that the genes including *ndhLSM* and *6hlno* in *A. nicotinovorans* pAO1, *hspB, hpo, nfo, ami*, and *iso* in *Pseudomonas putida* S16 all had hits in one 31-kb scaffold of the genome of strain TY, demonstrating a compact arrangement in this scaffold (Figure 1). Among these putative genes were two genes, *ndpA*_*S*_ (54%) and *ndpA*_*L*_ (37%), which showed amino acid sequence similarities to *ndhS* and *ndhL*, respectively. *ndpB* (43%) displayed similarities to *6hlno*, and *ndpH* (74%), *ndpF* (64%), *ndpE* (72%), *ndpG* (68%), and *ndpD* (54%) all shared considerable similarities with *iso, nfo, hpo, ami*, and *hspB*, respectively. This gene cluster was considered to be responsible for nicotine degradation and designated *ndp*. Until now, the underlying molecular mechanism has been the most well-studied in strain SJY1, excluding the enzyme that catalyzes the reaction from 6HPON to HSP (Yu et al., 2014, 2015); this enzyme was recently found in strain S33 (Li et al., 2016). Analysis of the whole genome sequence of strains SJY1 (accession number AZRT00000000) and S33 (CP014259.1 and CP014260.1; Yu et al., 2014; Li et al., 2016) showed that the gene organization and protein sequences of the nicotine-degrading gene clusters in these two strains were nearly identical (Figure 1). However, the sequence identity of *ndp* was significantly lower at the amino acid sequence (45–80%) and nucleotide sequence (56–75%) levels compared with strains SJY1 and S33. Moreover, the genetic organization of *ndp* was clearly different compared with the *vpp* gene cluster in strains SJY1 and S33. There was almost a 20-kb interval sequence between 6-hydroxypseudooxynicotine oxidase and maleamate amidase in the *vpp* gene cluster. In the 20-kb interval sequence, there were 6 and 11 mobile element protein genes in strains S33 and SJY1, respectively. However, the *ndp* gene cluster exhibited a compact arrangement without too much irrelevant sequence and no mobile element protein genes between the nicotine-degrading genes. In addition, we inferred that the undiscovered 6-hydroxypseudooxynicotine oxidase in SJY1 occurred at the same position as *pno* (Figure 1) due to their identical coding sequence.

### Transcription levels of putative nicotine-degrading genes in *ndp* are upregulated by nicotine

**Figure 2:**
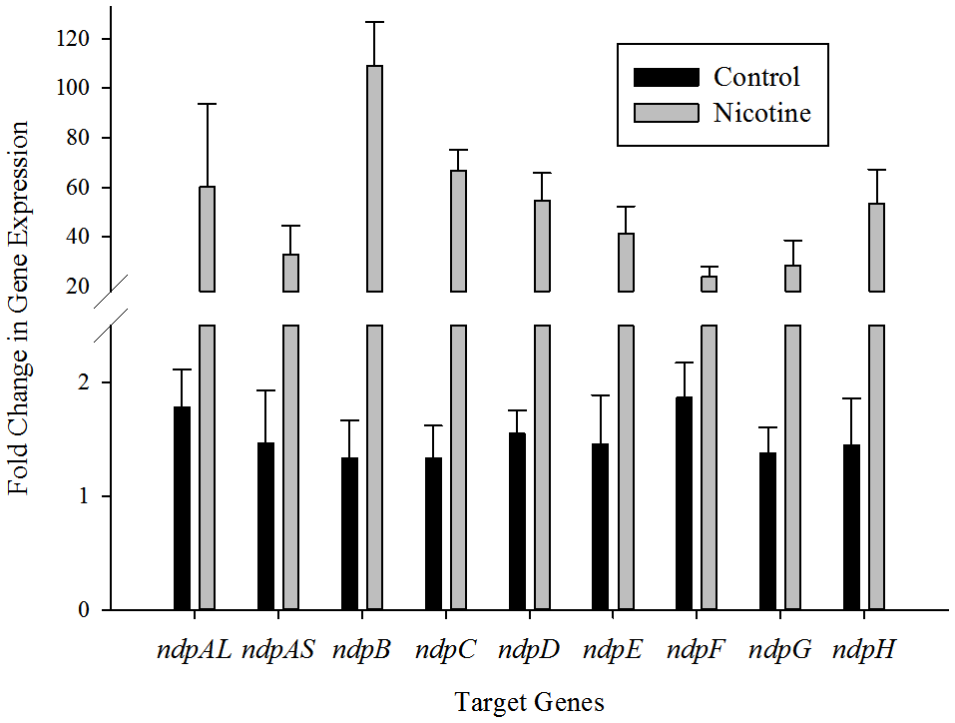
**Transcriptional analysis of the ***ndp*** gene cluster**. RT-qPCR analysis of target gene transcripts produced in *Sphingomonas melonis* TY grown with (gray bars) or without (black bars) nicotine. The expression levels of the *ndp* genes were normalized to the 16S rRNA and are expressed as fold changes relative to the expression level in cells. The results presented in these histograms are the means of three independent experiments, and error bars indicate the standard error.

To elucidate the correlation between nicotine degradation and putative nicotine-degrading genes in *ndp*, the mRNA expression levels of nine putative target genes involved in the nicotine degradation of *S. melonis* TY were estimated using RT-qPCR and the 2^ΔΔCT^ method with or without nicotine supplementation in the growth medium. The results showed that the levels of these genes were significantly upregulated in the presence of nicotine (Figure 2), suggesting that the transcription of these putative nicotine-degrading genes in *ndp* was induced by nicotine or other nicotine degradation intermediates. As shown in Figure 2, *ndpA*_*L*_, *ndpB, ndpC, ndpD*, and *ndpH* displayed more than 50-times higher than the levels of the corresponding genes in the control group, while *ndpA*_*S*_, *ndpF*, and *ndpG* exhibited relatively lower levels of transcription.

### NdpA catalyzes the reaction from nicotine to 6HN

**Figure 3:**
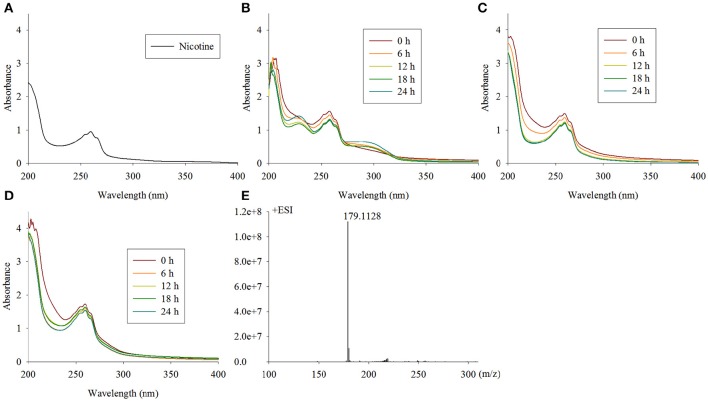
**Intermediate accumulated by TYΔ***ndpA***_***L***_ and biotransformation of nicotine by ***Sphingomonas-ndpA***, KT-***ndpA*** and KT-***ndpA***_**plus**_. (A)** Nicotine with a maximum absorbance at 259 nm by UV scanning was accumulated by TYΔ*ndpA*_*L*_; **(B–D)** biotransformation of nicotine by *Sphingomonas*-*ndpA*, KT-*ndpA*, and KT-*ndpA*_*plus*_, shows that product was only formed in *Sphingomonas*-*ndpA* (product should be 6-hydroxynicotine with a maximum absorbance at 232 and 295 nm); **(E)** LC-MS analysis of the 6HN produced in **(B)**.

The first step in the VPP is hydroxylation of the C6 at the pyridine ring to generate 6HN. The mutant TYΔ*ndpA*_*L*_ lost the ability to grow on nicotine, and after complementation of the full-length gene, it regained its ability to degrade nicotine. A biotransformation test with TYΔ*ndpA*_*L*_ revealed that no intermediate was accumulated by TYΔ*ndpA*_*L*_, according to UV scanning with a maximum absorbance at 259 nm (equivalent to nicotine) and LC-MS analysis. Additionally, TYΔ*ndpA*_*L*_ could transform 6HN, 6HPON and HSP but not nicotine, as expected (Table 1). The phenotypic traits of TYΔ*ndpA*_*L*_ suggested that NdpA was responsible for catalyzing the reaction from nicotine to 6HN. Heterologous expression of NdpA in *P. putida* KT2440 provided negative results, irrespective of the presence of pRK415*ndpA* or pRK415*ndpA*_*plus*_. NdpA was successfully expressed in *Sphingomonas aquatilis* JSS7^T^, and a decrease in the nicotine substrate resulted in the gradual production of the product 6HN (Figure 3).

**Table 1:** **Characteristics of mutant and complementary strains**.

			**Biotransformation**			
**Strains**	**Growth with nicotine**	**Accumulated intermediate metabolite**	**Nicotine**	**6HN**	**6HPON**	**HSP**
TY	+^a^	NA^b^	+	+	+	+
TYΔ*ndpA_*L*_*	−^c^	Nicotine	−	+	+	+
TYΔ*ndpB*	−	6HN	+	−	+	+
TYΔ*ndpC*	−	6HPON	+	+	−	+
TYΔ*ndpD*	−	HSP	+	+	+	−
TYΔ*ndpA_*L*_*(pRK415-*ndpA_*L*_*)	+	NA	NA	NA	NA	NA
TYΔ*ndpB*(pRK415-*ndpB*)	+	NA	NA	NA	NA	NA
TYΔ*ndpC*(pRK415-*ndpC*)	+	NA	NA	NA	NA	NA
TYΔ*ndpD*(pRK415-*ndpD*)	+	NA	NA	NA	NA	NA

a *means have corresponding ability*;

b *means not applicable*;

c *means have no corresponding ability*.

### The *ndpB* gene encodes 6-hydroxynicotine oxidase

The second enzymatic step in the VPP of nicotine degradation is the transformation of 6HN to 6-hydroxy-N-methylmyosmine (6HMM). After the disruption of *ndpB*, the mutant strain TYΔ*ndpB* lost the ability to grow on nicotine, and complementation restored nicotine-degrading ability similar to the wild-type strain. A biotransformation test illustrated that the intermediate that accumulated in TYΔ*ndpB* was 6HN, according to UV scanning (maximum absorbances at 232 and 295 nm) and LC-MS analysis. It also showed that TYΔ*ndpB* could effectively transform nicotine, 6HPON and HSP (Table 1). The phenotypic traits of TYΔ*ndpB* and the complementary experiment suggested that *ndpB* catalyzed the transformation from 6HN to 6HMM. Recombinant NdpB formed in inclusion bodies following the overexpression in *E. coli* BL21(DE3). Additionally, renaturation of NdpB failed in the absence of active enzyme.

To avoid inclusion body formation and obtain soluble protein, pET-22b(+) was selected for secretive expression of NdpB, and the expression host was changed to Origami B(DE3). Unfortunately, the NdpB still formed in inclusion bodies (data not shown).

**Figure 4:**
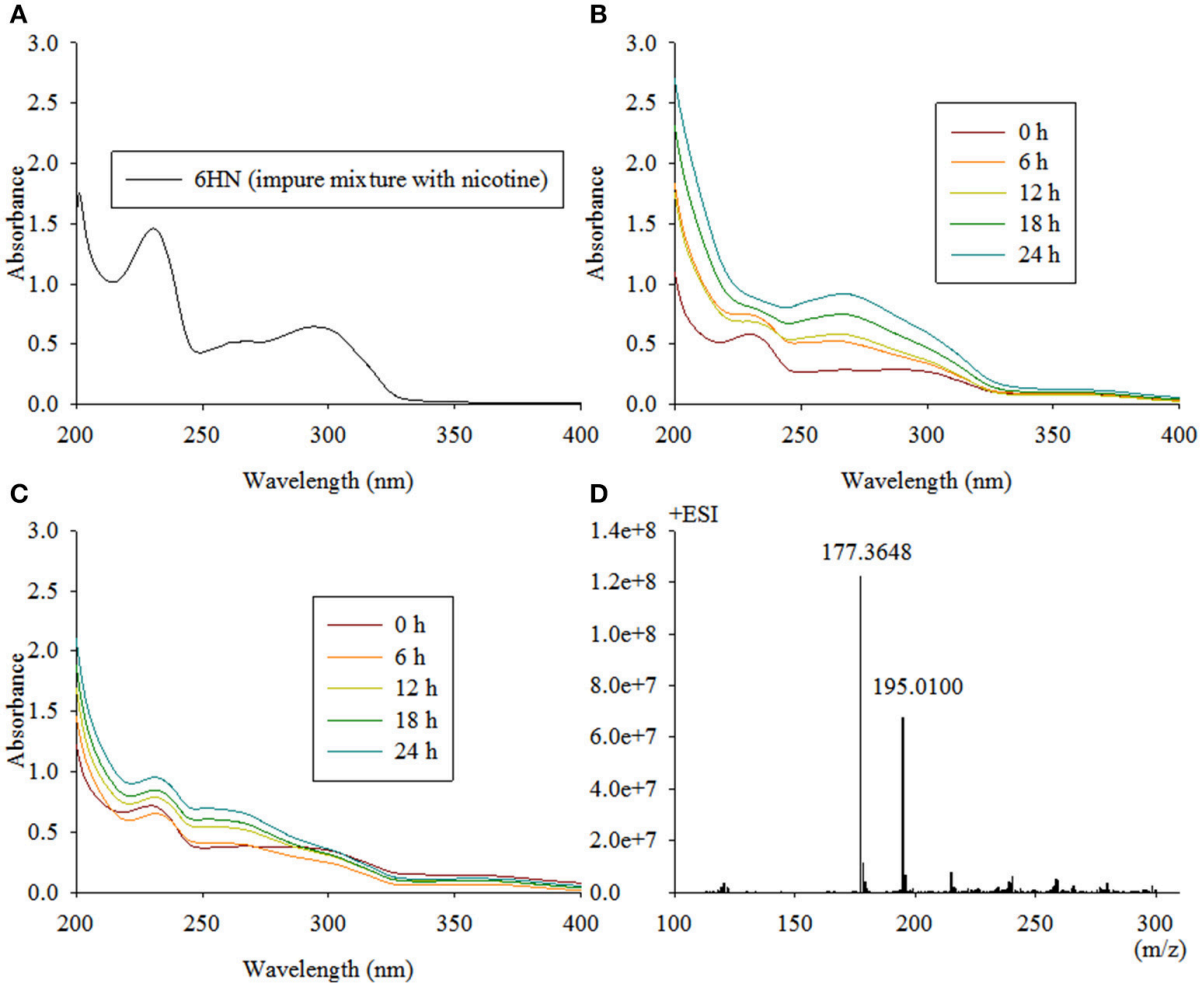
**Intermediate accumulated by TYΔ***ndpB*** and biotransformation of 6HN by Sphingomonas-***ndpB*** and KT-***ndpB***. (A)** 6HN with a maximum absorbance at 232 and 295 nm by UV scanning was accumulated by TYΔ*ndpB*; **(B,C)** biotransformation of 6HN by *Sphingomonas*-*ndpB* and KT-*ndpB*, respectively, showed that no product was formed in strain KT-*ndpB*, whereas *Sphingomonas*-*ndpB* exhibited catalytic activity based on 6-hydroxy-N-methylmyosmine formation. LC-MS analysis of the product shown in **(D)**, and 6HMM formed by purified NdpB demonstrates the same peak as in **(D)**.

**Figure 5:**
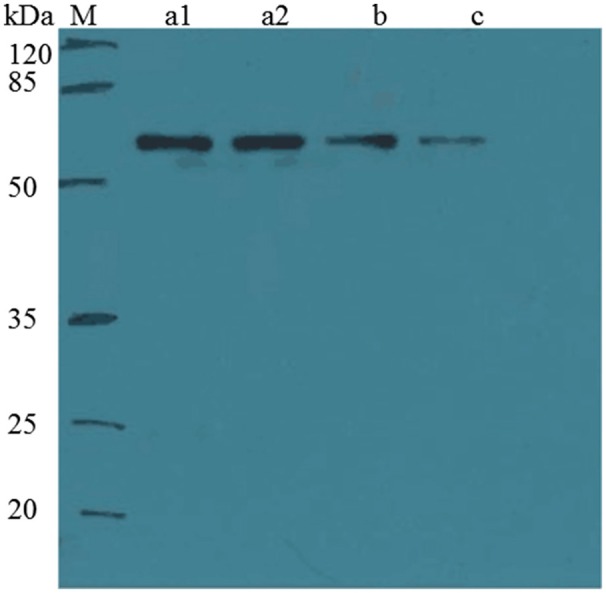
**Western blot analysis of purified NdpB expressed in ***Sphingomonas*** TYΔ***ndpB*****. Western blot analysis of the samples using a His tag-specific antibody. The contents of the lanes are as follows: M, marker; a1, original sample; a2, parallel sample with a2; b, 10-fold dilution of the original sample; and c, 100-fold dilution of the original sample.

Because *E. coli* strains were not suitable for the expression of NdpB, we placed pRK415*ndpB* in *Sphingomonas aquatilis* JSS7^T^ and detected the catalysis of 6HN. Interestingly, NdpB expressed in *Sphingomonas aquatilis* JSS7^T^ was active and transformed 6HN to 6HPON, whereas active expression in *P. putida* KT2440 failed (Figure 4). We obtained 100 μg of purified NdpB from the expression of NdpB in strain TYΔ*ndpB*, and the western blot analysis is shown in Figure 5. The results suggested that the correct protein was acquired with the expected molecular weight and with high purity. However, very weak activity was detected at the primary stage when conducting the enzymatic reaction, and the product was found to be 6HMM by LC-MS (Figure 4).

### NdpC is responsible for the transformation of 6HPON

**Figure 6:**
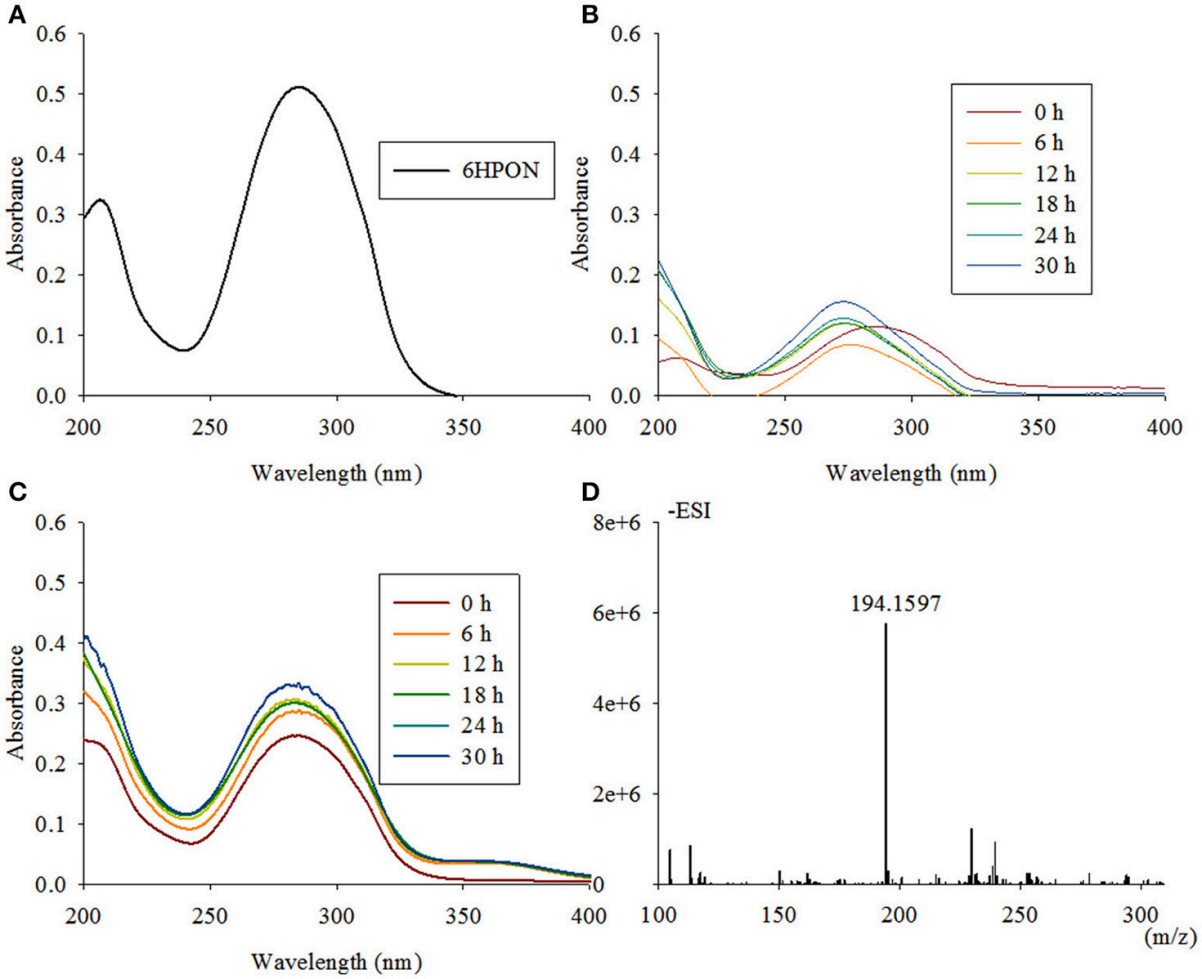
**Intermediate accumulated by TYΔ***ndpC*** and biotransformation of 6HPON by ***Sphingomonas-ndpC*** and KT-***ndpC***. (A)** 6HPON with a maximum absorbance at 289 nm was accumulated by TYΔ*ndpC* based on UV scanning; **(B,C)** biotransformation of 6HPON by *Sphingomonas*-*ndpC* and KT-*ndpC* showed that no product was formed in strain KT-*ndpC*, whereas *Sphingomonas*-*ndpC* had catalytic activity based on the formation of 6-hydroxy-3-succinoyl-pyridine. LC-MS analysis of the product shown in **(D)**.

The reaction from 6HPON to HSP is one of the key steps in the VPP, causing the degradation pathway that starts at the PD to switch to the PRL. TYΔ*ndpC*, as well as TYΔ*ndpB* and TYΔ*ndpA*_*L*_, lost the ability to grow with nicotine as the sole carbon and nitrogen source. After gene complementation, the complementary strain recovered the ability to degrade nicotine. A biotransformation test showed that 6HPON was the accumulated intermediate produced by TYΔ*ndpC*, according to UV scanning (maximum absorbance at 289 nm, pH < 8) and LC-MS analysis. Additionally, TYΔ*ndpC* could convert nicotine, 6HN and HSP (Table 1). The phenotypic characteristics of TYΔ*ndpC* indicated that *ndpC* might be responsible for transforming 6HPON to HSP. Heterologous expression of *ndpC* in *P. putida* KT2440 provided negative results, whereas the transfer of *Sphingomonas aquatilis* JSS7^T^ with *ndpC* provided the ability to transform 6HPON into HSP (Figure 6). However, it was considered that there was a 6-hydroxy-3-succinoylsemialdehyde-pyridine formed between 6HPON and HSP (Ma et al., 2013), but this dehydrogenation step may be performed by another non-specific semialdehyde dehydrogenase in strain.

### NdpD is 6-hydroxy-3-succinoyl-pyridine 3-monooxygenase

**Figure 7:**
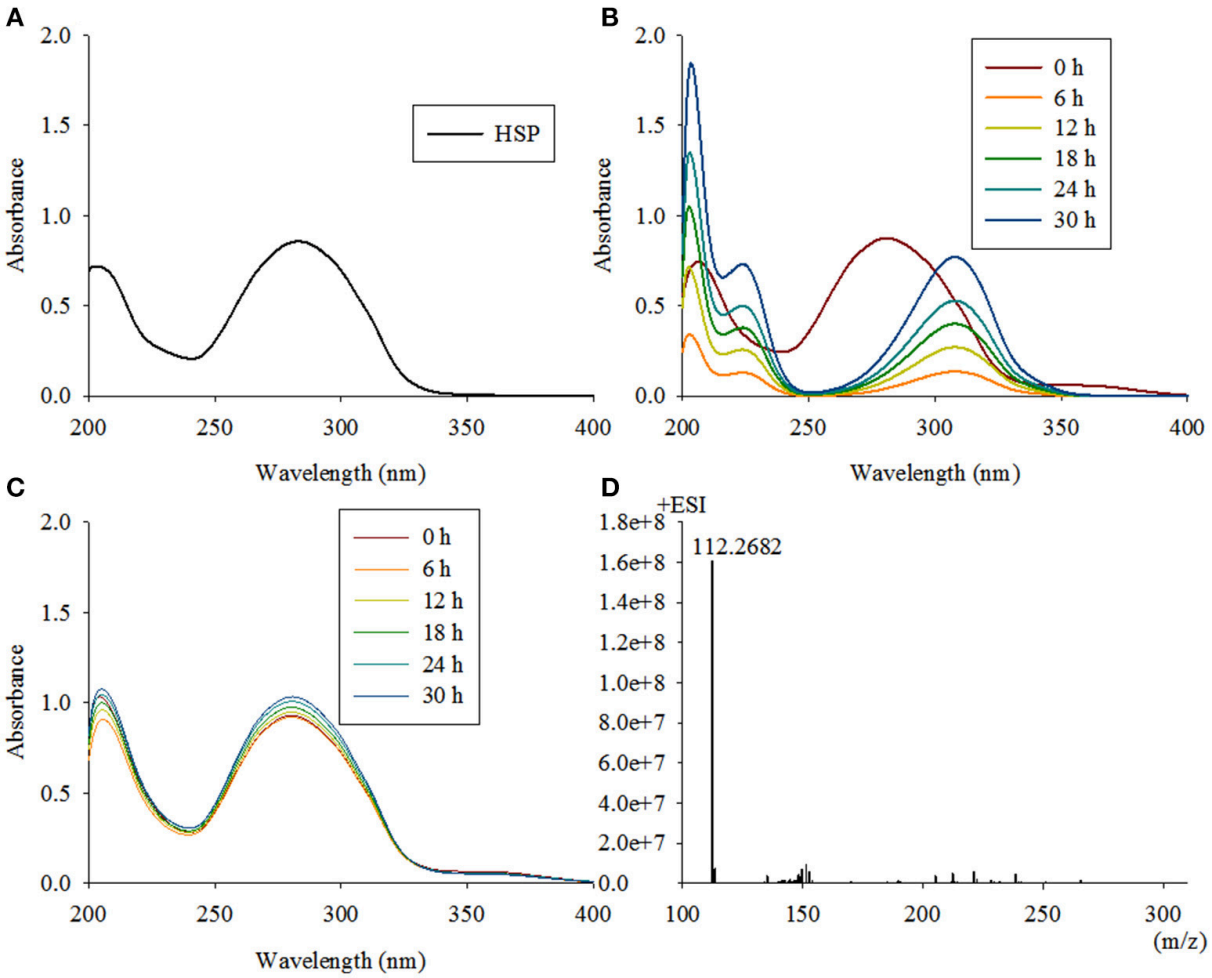
**Intermediate accumulated by TYΔ***ndpD*** and biotransformation of 6-hydroxy-3-succinoyl-pyridine by ***Sphingomonas-ndpD*** and KT-***ndpD***. (A)** 6-hydroxy-3-succinoyl-pyridine with a maximum absorbance at 276 nm was accumulated by TYΔ*ndpD* based on UV scanning; **(B,C)** biotransformation of 6-hydroxy-3-succinoyl-pyridine by *Sphingomonas*-*ndpD* and KT-*ndpD* showed that no product was formed in strain KT-*ndpC*, whereas *Sphingomonas*-*ndpC* displayed catalytic activity based on 2, 5-dihydroxy-pyridine formation. LC-MS analysis of the product shown in **(D)**.

The fourth enzymatic step in the VPP is the formation of 2, 5-dihydroxypyridine (2, 5-DHP), which is an intermediate that is generated during the degradation of many pyridine derivatives by aerobic microorganisms (Yao et al., 2013). The mutant strain TYΔ*ndpD* lost the ability to grow on nicotine, which was restored after gene complementation. A biotransformation test with TYΔ*ndpD* revealed an accumulation of the intermediate HSP, according to UV scanning with a maximum absorbance at 276 nm and LC-MS analysis. Additionally, it was anticipated that TYΔ*ndpD* could catalyze the transformations of nicotine, 6HN, and 6HPON (Table 1). Recombinant NdpD was primarily overexpressed in *E. coli* BL21(DE3) as a C-terminal His_6_-tagged fusion protein in pET-28a(+). A band at an apparent molecular mass of 45.1 kDa was detected by SDS-PAGE in the precipitate (data not shown), which corresponded to the molecular weight of His_6_-tagged NdpD. To express soluble NdpD, the N-terminal His_6_-tagged fusion protein in pET-28a(+) was selected for expression in *E. coli* BL21(DE3). Fortunately, a small amount of soluble NdpD protein in supernatant (data not shown), but no product formed during detection of the crude enzyme activity. However, heterologous expression of NdpD in *P. putida* KT2440 provided negative results, but the ability of NdpD to catalyze the transformation of HSP to 2, 5-DHP was successfully detected in *Sphingomonas aquatilis* JSS7^T^ (Figure 7).

### *ndp* has different genetic characteristics compared with *vpp*

**Figure 8:**
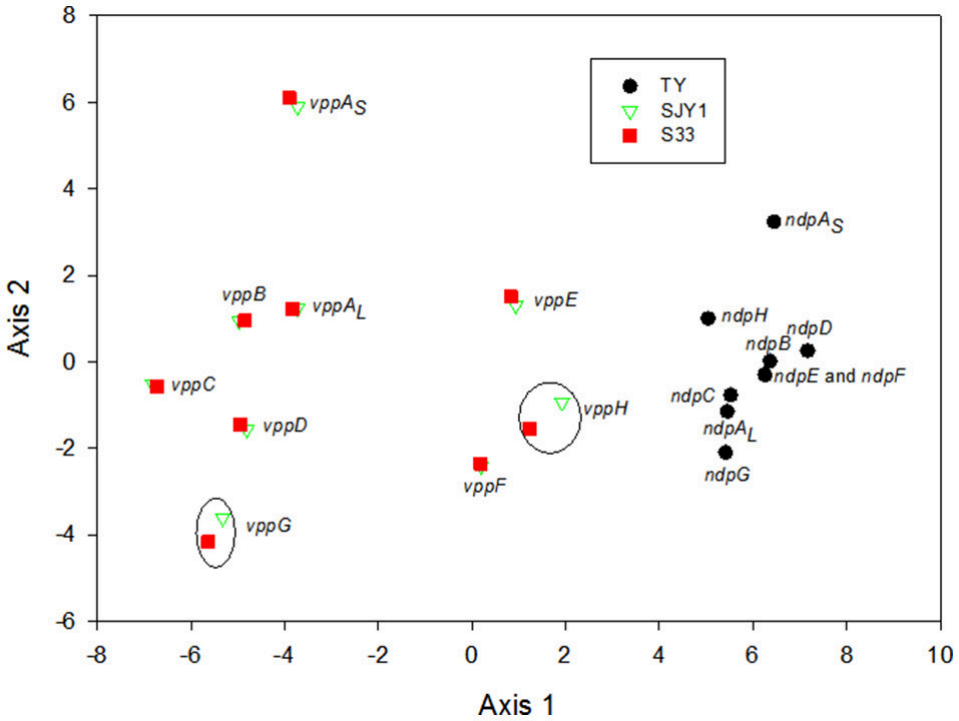
**Principal component analysis of the codon usage of nicotine-catalyzing genes in strains TY, SJY1, and S33**. Black dots, genes from strain TY; red squares, genes from strain S33; green triangles, genes from strain SJY1. Most of the homologous genes in SJY1 and S33 were overlapping, and the genes in black circles were homologous genes in strains SJY1 and S33. *ndpE* and *ndpF* almost overlapped with each other in strain TY.

The G+C content of nicotine-degrading gene clusters in TY, SJY1, and S33 compared with the G+C content of their genomes revealed no significant differences (Table 2). The RSCU analysis showed that strains SJY1 and S33 have nearly the same codon usage bias as well as %G3s+C3s for each corresponding nicotine-degrading gene, whereas strain TY has a distinct RSCU value and significantly higher %G3s+C3s (Figure 8, Table 2). A BLASTp search revealed that homologous genes of nicotine-degrading genes in strain TY are present in *Sphingomonas* sp. Ant20, *Sphingobium xenophagum, Sphingobium chungbukense, Sphingobium* sp. KK22, *Sphingopyxis* sp. H080, and other closely related species, all of which belong to the family Sphingomonadaceae. While homologous genes of nicotine-degrading genes in SJY1 and S33 are distributed in much more distantly related taxa, such as species of *Sphingomonas* and *Sphingobium* for strain SJY1 and species of *Rhizobium, Shinella, Paramesorhizobium, Sphingopyxis*, and *Sphingomonas* for strain S33, all the species that displayed similarity to strain SJY1 or S33 belonged to a different order or class. Several clusters of mobile element protein genes were found in nicotine-degrading genes or adjacent sequences of strain SJY1 and S33, but none were found in *ndp*. In addition to all the necessary nicotine metabolism genes, a transmembrane protein was encoded by *ndp* and was probably related to the transport of nicotine or other critical metabolite of nicotine degradation in strain TY. The mean ENC in TY was 35.67, and the mean ENC in *ndp* was 34.34. In general, based on the clustered genetic organization and integrity of *ndp* and considering the above results, *ndp* had different genetic characteristics compared with *vpp*, and *vpp* in strains SJY1 and S33 appeared to evolve from HGT. Nevertheless, these results didn't rule out the possibility that *ndp* was originated from HGT. On the contrary, from the results of RSCU analysis of 3867 genes in draft genome of strain TY (Figure S2), it seems that there was a considerable possibility that some genes in *ndp* were originated from HGT.

**Table 2:** **Comparison of the G+C content and nucleotide composition of the genome, gene cluster, and encoded genes**.

**TY**	**%G+C**	**%G3s+C3s**	**SJY1**	**%G+C**	**%G3s+C3s**	**S33**	**%G+C**	**%G3s+C3s**
Genome	67.10	/	Genome	54.2		Genome	59.16	/
Gene cluster	66	/	Gene cluster	55.70	/	Gene cluster	55	/
*ndpAL*	67.21	85.93	*vppAL*	55.46	60.9655	*ndhA*	55.28	60.68
*ndpAS*	64.90	87.5	*vppAS*	56.41	59.7315	*ndhB*	56.19	59.06
*ndpB*	66.39	88.18	*vppB*	53.34	56.5012	*hno*	53.42	56.97
*ndpC*	65.73	83.38	*vppC*^a^	53.22	50.463	*pno*	53.32	50.77
*ndpD*	65.89	87.63	*vppD*	53.74	56.3342	*hsh*	53.74	56.06
*ndpE*	65.90	85.49	*vppE*	58.79	73.27044	*S33vppE*	58.69	72.95
*ndpF*	67.51	83.26	*vppF*	64.62	71.6599	*S33vppF*	64.62	71.65
*ndpG*	66.82	82.5	*vppG*	55.76	55.3922	*S33vppG*	55.29	53.69
*ndpH*	65.95	84.61	*vppH*	62.73	77.1739	*S33vppH*	61.06	75.74

a *Gene name in gray text means they were putative by analysis this work*.

## Discussion

Microbial degradation plays an important role in the elimination of nicotine pollution in the environment. In this study, a novel nicotine degradation gene cluster, *ndp*, was identified in strain TY, which degraded nicotine efficiently via the VPP. The molecular mechanisms of nicotine degradation in this strain and the functions of four genes, *ndpA, ndpB, ndpC*, and *ndpD*, were studied. NdpA is responsible for the hydroxylation of nicotine to 6HN, NdpB catalyzes the conversion of 6HN to 6HMM, NdpC transforms 6HPON to HSP, and NdpD converts HSP to 2, 5-DHP. Elucidation of the molecular mechanism of nicotine degradation in strain TY provides a new resource for industrial applications and the management of nicotine-polluted environments.

Analysis based on *vpp* and *ndp* shows that the homologous genes of nicotine-degrading genes show a scattered phylogenetic distribution in strains SJY1 and S33, whereas the homologous genes of nicotine-degrading genes in TY are distributed all in closely related taxa and species. There were no distinct difference in G+C content between the nicotine-degrading gene cluster and the genome in strains S33 and SJY1; however, this may be due to genetic homogenization during long-term evolution following HGT of these genes. Moreover, there were no distinct differences in the G+C content of the genome and cluster in strain TY. The RSCU values are useful for comparing codon usage among genes or sets of genes (Andersson and Sharp, 1996). The RSCU value and percentage of G3s+C3s of nicotine-degrading genes in strains SJY1 and S33 were highly consistent, despite the very distant relationship of these strains. In contrast, the nicotine-degrading genes in strain TY had a remarkably different codon usage bias and nucleotide composition compared with strains SJY1 and S33. Additionally, the regions contiguous to the genes that were confirmed to be horizontally transferred were observed for the nicotine-degrading gene cluster in strains SJY1 and S33. However, there was no trace of mobile elements in *ndp* in strain TY, may be due to the deletion of intervening genes (such as mobile elements) that do not provide a selectable function in certain environments, according to the Selfish Operon Model of gene clustering in prokaryotes and eukaryotes, thus facilitating the evolution of clustered, energy efficient, dissemination (both by vertical transmission and by horizontal transfer) and functional gene clusters (Lawrence and Roth, 1996; Lawrence, 1999). Meanwhile, it may be due to the genome rearrangement of orthologous pathway (Periwal and Scaria, 2014).

Substrate transport-related transporter genes are often contiguous with catabolism genes in the bacterial genome to save energy and respond rapidly to environmental stress. For example, MhbT, a specific transporter for the uptake of 3-hydroxybenzoate in *Klebsiella pneumoniae* M5a1, is encoded by a cluster, *mhbRTDHIM*, containing enzymes that convert 3-hydroxybenzoate to pyruvate and fumarate via gentisate and a gene activator, *mhbR* (Xu et al., 2012). MhpT, a 3-(3-hydroxyphenyl) propionate (3HPP) transporter in *Escherichia coli* K-12, is encoded by cluster *mhpRABCDFET*, and the other genes in this cluster are responsible for regulatory functions and 3HPP catabolism (Ferrández et al., 1997; Torres et al., 2003; Xu et al., 2013). GabP_Cg_, a γ-aminobutyric acid (GABA) transporter, is adjacent to succinic semialdehyde dehydrogenase (GabD) and GABA oxoglutarate aminotransferase (GabT) in the genome of *Corynebacterium glutamicum* (Zhao et al., 2012). Interestingly, we identified a transmembrane protein, NdpT, in *ndp* (Figure 1). Additionally, *ndpT* was tentatively proposed to encode a potential transporter involved in the uptake of nicotine, its metabolic intermediates, or both. Furthermore, this gene was absent in strains SJY1 and S33. Combining the scattered phylogenetic distribution of nicotine-degrading genes in strains SJY1 and S33, the concentrated structure of nicotine-degrading genes in strain TY and the above-described results, we considered that the *vpp* in strains SJY1 and S33 resulted directly from HGT and that *ndp* was the gene cluster result from genome rearrangement of orthologous pathways or from Selfish Operon Model, but with a high degree of homogenization. The specificity of the *ndp* gene cluster may explain why *P. putida* KT2440 is unable to express active nicotine-degrading genes in strain TY, in contrast to *Sphingomonas aquatilis* JSS7^T^. However, VppA can be successfully expressed in *P. putida* KT2440 (Yu et al., 2015), further supporting the differences between *ndp* and *vpp* clusters.

**Figure 9:**
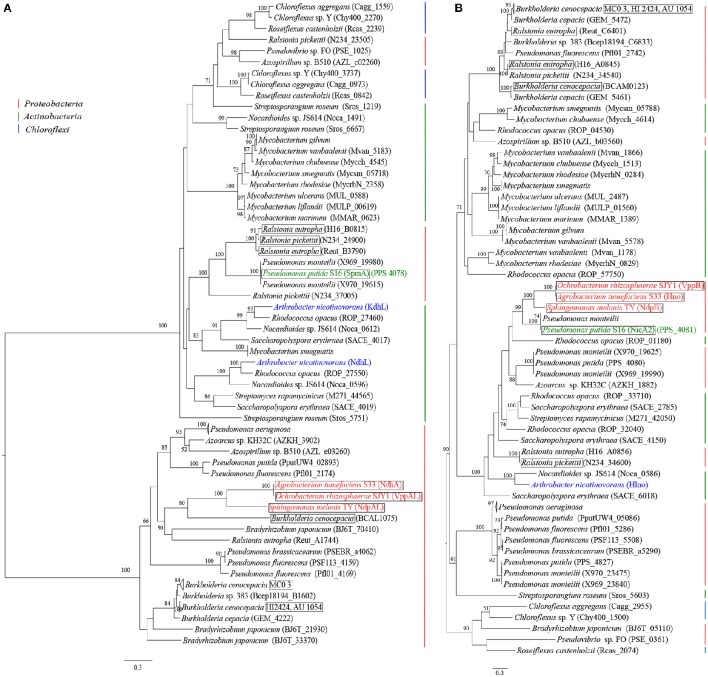
**Phylogenetic trees of the homologs of the hydroxylase of the pyridine ring and dehydrogenase of the pyrrolidine ring. (A)** Phylogenetic tree of the homologs of the hydroxylase of the pyridine ring; **(B)** Phylogenetic tree of the homologs of the dehydrogenase of the pyrrolidine ring. The trees were constructed using homologs of the corresponding enzymes from 50 bacterial genomes (the information of these homologs is listed in Table S4). The colored text denotes reported nicotine-degrading strains, and the strains marked by a square frame have linked *ndpHFEG*.

The evolutionary relationships analysis showed that the nicotine hydroxylase large subunit (NdpA_*L*_) in the VPP, 3-succinoylpyridine monooxygenase *alpha* subunit (SpmA) in the PRL, and the nicotine dehydrogenase (NdhL) and ketone dehydrogenase (KdhL) in the PD belonged to the same protein family (Figure 9). Evidently, the phylogenies of the NdpA_*L*_ and NdpB proteins were completely different (Figure 9). This result indicated that although they were recruited in the same metabolic pathway, they had different evolutionary patterns. In particular, NdpA_*L*_ resided in two clear phylogenetic clades. Interestingly, although the VPP and PRL pathways were more similar in terms of metabolic mechanisms, NdpA_*L*_ and SpmA, respectively, belonged to different protein subfamilies with potentially different origins that had undergone convergent evolution. However, 6-hydroxy-L-nicotine oxidase, NdpB, and NicA2 (PRL) had relative closer evolutionary relationships. According to the patchwork hypothesis, the ancient gene could have encoded a primitive enzyme with low substrate specificity to catalyze distinct but similar reactions, and it was recruited into various biological pathways (Jensen, 1976; Lazcano and Miller, 1996). Our results based on the phylogenies of the NdpA_*L*_ and NdpB proteins demonstrated that the VPP and PRL pathways recruited different genotypes. We also found that the *ndpHFEG* genes were linked in some strains (Figure 9). The linkages among these four genes might represent a common genetic structure in the VPP and PRL pathways.

In the PD, we found that KdhL and NdhL had a closer evolutionary relationship and that these proteins can also be identified in other closely related species such as *Rhodococcus opacus, Nocardioides* sp. JS614 and *Saccharopolyspora erythraea*. We thought that the PD would be distributed in Gram-positive bacteria and that the VPP and PRL pathways would be distributed in Gram-negative bacteria (Table S3). To further study this hypothesis, we evaluated the gram staining of the strains with of *ndpHFEG*. As expected, the linked homologs of *ndpHFEG* identified by BLAST searches against 2806 predicted prokaryotic proteomes were in Gram-negative strains (Table S3). Moreover, one of the three degradation pathways of nicotinic acid gone through 2, 5-DHP was only found in Gram-negative strains (Table S3). Surprisingly, we found that *Streptomyces rapamycinicus* might have an entire nicotine-degrading pathway that is not a PD. Although *S. rapamycinicus* only had linked *ndpHFG* genes, one protein exhibited 34.02% similarity to the NdpE of *S. melonis* TY. However, further experimental evidence is needed to support this result. In summary, in terms of both evolution and metabolic mechanism, the VPP was more similar to the PRL. The linkage of *ndpHFEG* genes shared by both pathways indicated that these two pathways might have the same origin; however, variants occurred in some bacteria.

In addition to the common reactions of hydroxylation at C6 of the pyridine ring and dehydrogenation at the C2–C3 bond of the pyrrolidine ring, there are three other similar enzyme-catalyzed reactions (cleavage of the pyrrolidine residue in the pyridine ring, deamination and dehydrogenation of the pyrrolidine residue) and one step of autohydrolysis of the pyrrolidine ring that are, in fact, shared by the VPP, PRL, and PD pathways (Wang et al., 2012). Moreover, the codon usage of representative strains of the VPP, PRL, and PD pathways was distinct (Figure S1). We speculated that a series of ancient genes could have encoded primitive enzymes with low substrate specificity to catalyze distinct but similar reactions, and these genes were recruited to various biological pathways to form distinct pathways over long-term evolution, according to the patchwork hypothesis (Jensen, 1976; Lazcano and Miller, 1996). Together with the analysis of evolution and relationship of the metabolic mechanisms of the VPP and PRL pathways described above, it was reasonable to consider that the VPP, PRL, and PD pathways may have experienced independent but interrelated evolutionary events.

The hypothesis that the PD is distributed in Gram-positive bacteria and that the VPP and PRL pathway is distributed in Gram-negative bacteria was based on current research and was an innovative inference. This hypothesis can be verified by a simple experiment that is theoretically feasible. The representative intermediate product of the VPP and PRL pathways can be used as a sole carbon and/or nitrogen source to screen Gram-positive bacterial growth, and conversely, we can screen the growth of Gram-negative bacteria on the pyridine pathway-specific metabolite. After analyzing the intermediates produced in the pyridine, VPP and PRL pathways, we considered selecting 2, 5-DHP as the representative metabolic product in the VPP and PRL pathways and 2, 6-dihydroxy-pseudohydroxypyridine as the product in the PD. The 2, 5-DHP is an intermediate product that is shared by the nicotine and nicotinic acid degradation pathways (Jiménez et al., 2008), and all the strains collected using the 2, 5-DHP pathway were Gram-negative, supporting our speculation. A second strategy to support or dismiss our hypothesis is to examine whether certain homologs of *ndpHFEG* are active in specific Gram-positive strains. If these homologs become active, then our hypothesis is incorrect, and natural Gram-positive strain that utilize the VPP or PRL pathway will eventually be obtained. Conversely, our hypothesis is reasonable and has research value. Of course, the Gram-positive strain *S. rapamycinicus*, which carries the homologs of *ndpA*_*L*_, *ndpB, ndpE*, and *ndpHFG*, can be assessed for nicotine degradation ability and the associated pathway. Strain, *Pusillimonas* sp. T2, which contained nicotine metabolites from both the VPP and PD pathways (2, 6-dihydroxypyridine), may be explained by the substrate ambiguity of nicotine hydroxylase. After hydroxylation of C6 of the pyridine ring, some of the 6HPON intermediate was hydroxylated again at C2 of pyridine to generate 2, 6-dihydroxy-pseudooxynicotine, which was then hydroxylated by a non-specific C-C hydrolase to produce 2, 6-dihydroxypyridine. Otherwise, the portion of the *vpp* cluster in *Pusillimonas* sp. T2 was different with what we were discussed about. Four nicotine degradation intermediates produced by Gram-negative *Achromobacter nicotinophagum* have been mentioned above and are related to the PD (6HN), PRL (pseudooxynicotine, 3-succinoyl-pyridine and HSP), and VPP (6HN and HSP). We thought that this strain might contain a complete PRL and that due to the substrate ambiguity of the hydroxylase of pyridine ring, the strain might be capable of forming 6HN. On the other hand, this strain may contain both the PD and the PRL pathway.

In summary, we discovered a new nicotine degradation gene cluster, *ndp*, and characterized four genes that catalyze the first four enzymatic steps in the VPP in *S. melonis* TY. These results provide relatively comprehensive evidence for the molecular mechanism of nicotine degradation in *S. melonis* TY. We also formulated an inference that both the evolutionary features and metabolic mechanisms of the VPP were more similar to the PRL. These findings provide a deeper understanding of the evolution of nicotine metabolism in *Sphingomonas*. The hypothesis that the PD is distributed in Gram-positive bacteria while the VPP and PRL pathways are distributed in Gram-negative bacteria based on the result of comprehensive homologs searching and phylogenetic tree construction and that the evolutionary relationships among the VPP, PRL and PD pathways will provide critical information that will improve our understanding of the evolution of nicotine-degrading gene clusters.

## Ethics statement

This article does not contain any studies with human participants or animals performed by any of the authors.

## Author contributions

Performed experiments: HW and XZ. Analyzed data: HW, XZ, JQ, and LS. Conceived and designed experiments, wrote the paper and approved the final manuscript: All authors.

## Funding

This work was financially supported by the Natural Science Foundation for Distinguished Young Scholars of Zhejiang Province (LR14D030001), and the National Natural Science Foundation of China (No. 31170115 and 31422003).

### Conflict of interest statement

The authors declare that the research was conducted in the absence of any commercial or financial relationships that could be construed as a potential conflict of interest.
