# Supplementary material for: Characterization of a Novel Nicotine Degradation Gene Cluster ndp in Sphingomonas melonis TY and Its Evolutionary Analysis
Source: Front Microbiol. 2017 Mar 9;8:337. doi: 10.3389/fmicb.2017.00337 (PMC5343071; doi:10.3389/fmicb.2017.00337)
Supplement: Supplementary file 1 [file Table1.DOCX]

**Table S1** Strains and plasmids used in this study

| Strain or plasmid | Relevant characteristics | Reference or source |
| --- | --- | --- |
| Strains |  |  |
| *Escherichia coli* |  |  |
| DH5α | *supE44 lacU169*(φ80dlacZΔM15)*hsdR17 recA1 endA1 gyrA96Δthi relA1* | (Woodcock et al. 1989) |
| BL21(DE3) | F^-^ *ompT hsdS_B_*  (*r_B_*^-^ *m_B_*^-^)*gal dcm lacY1*(DE3) | Transgen |
| BL21(DE3)-*ndpB* | BL21(DE3) transformed with pET28a-*ndpB*, Kan^r^ | This study |
| BL21(DE3)-*ndpD*-C | BL21(DE3) transformed with pET28a-*ndpD*-C, Kan^r^ | This study |
| BL21(DE3)-*ndpD*-N | BL21(DE3) transformed with pET28a-*ndpD*-N, Kan^r^ | This study |
| Origami B(DE3) | F^-^ *ompT* *hsdS_B_*(*r_B_*^-^ *m_B_*^-^) *gal* *dcm* *lacY1* *ahpC* (DE3) *gor522*::Tn*10* (Tc^R^) *trx*B::kan | Transgen |
| OrigamiB(DE3)-*ndpB* | OrigamiB(DE3) transformed with pET22b-*ndpB*, Kan^r^, Tc^r^, Amp^r^ | This study |
| WM3064 | Donor strain for conjugation, 2,6-diaminopimelic  acid auxotroph: *thrB1*004 *pro* *thi* *rpsL hsdS*  *lacZ*ΔM15 RP4-1360 Δ(*araBAD*)*567*  Δ*dapA*1341::[*erm pir*(wt)] | (Dehio and Meyer 1997; Saltikov and Newman 2003) |
| *Sphingomonas* species |  |  |
| TY | Wild type, nicotine-degrading strain, G^-^, Amp^r^, Kan^s^, Tc^s^ | This study |
| TYΔ*ndpA_L_* | TY mutant with *ndpA_L_* gene replaced by kanamycin resistance gene from plasposon pTnMod-Okm, Amp^r^, Kan^r^ | This study |
| TYΔ*ndpB* | TY mutant with *ndpB* gene replaced by kanamycin resistance gene from plasposon pTnMod-Okm, Amp^r^, Kan^r^ | This study |
| TYΔ*ndpC* | TY mutant with *ndpC* gene replaced by kanamycin resistance gene from plasposon pTnMod-Okm, Amp^r^, Kan^r^ | This study |
| TYΔ*ndpD* | TY mutant with *ndpD* gene replaced by kanamycin resistance gene from plasposon pTnMod-Okm, Amp^r^, Kan^r^ | This study |
| TYΔ*ndpA_L_*（pRK415-*ndpA_L_*） | *ndpA_L_* gene was complemented by pRK415-*ndpA_L_* in TYΔ*ndpA_L_*, Amp^r^, Kan^r^, Tc^r^ | This study |
| TYΔ*ndpB*（pRK415-*ndpB*） | *ndpB* gene was complemented by pRK415-*ndpB* in TYΔ*ndpB*, Amp^r^, Kan^r^, Tc^r^ | This study |
| TYΔ*ndpC*（pRK415-*ndpC*） | *ndpC* gene was complemented by pRK415-*ndpC* in TYΔ*ndpC*, Amp^r^, Kan^r^, Tc^r^ | This study |
| TYΔ*ndpD*（pRK415-*ndpD*） | *ndpD* gene was complemented by pRK415-*ndpD* in TYΔ*ndpD*, Amp^r^, Kan^r^, Tc^r^ | This study |
| TYΔ*ndpB*-*ndpB_hi_*_s_ | TYΔ*ndpB* transformed with pRK415-*ndpB_his_*, Tc^R^ | This study |
| *Sphingomonas aquatilis* JSS7^T^ | Wild type, non-nicotine-degrading strain, G^-^, Tc^s^ | (Lee et al. 2001) |
| *Sphingomonas*-*ndpA* | *Sphingomonas aquatilis* transformed with pRK415-*ndpA*, Tc^r^ | This study |
| *Sphingomonas*-*ndpC* | *Sphingomonas aquatilis* transformed with pRK415-*ndpC*, Tc^r^ | This study |
| *Sphingomonas*-*ndpD* | *Sphingomonas aquatilis* transformed with pRK415-*ndpD*, Tc^r^ | This study |
| *Pseudomonas putida* species |  |  |
| KT2440 | Metabolically versatile saprophytic soil bacterium | (Nelson et al. 2002) |
| KT-*ndpA* | KT2440 transformed with pRK415-*ndpA*, Tc^r^ | This study |
| KT-*ndpA_plus_* | KT2440 transformed with pRK415-*ndpA_plus_*, Tc^r^ | This study |
| KT-*ndpB* | KT2440 transformed with pRK415-*ndpB*, Tc^r^ | This study |
| KT-*ndpC* | KT2440 transformed with pRK415-*ndpC*, Tc^r^ | This study |
| KT-*ndpD* | KT2440 transformed with pRK415-*ndpD*, Tc^r^ | This study |
| Plasmids |  |  |
| pTnMod-Okm | Source of kanamycin resistance gene | (Dennis and Zylstra 1998) |
| pEX18Tc | Gene knockout vector, oriT^+^, sacB^+^, Tc^r^ | (Hoang et al. 1998) |
| pEX18Tc-*ndpA_L_* | *ndpA_L_* gene knockout vector containing two DNA fragments homologous to the upstream and downstream regions of the *ndpA_L_* and kanamycin resistance gene from pTnMod-Okm | This study |
| pEX18Tc-*ndpB* | *ndpB* gene knockout vector containing two DNA fragments homologous to the upstream and downstream regions of the *ndpB* and kanamycin resistance gene from pTnMod-Okm | This study |
| pEX18Tc-*ndpC* | *ndpC* gene knockout vector containing two DNA fragments homologous to the upstream and downstream regions of the *ndpC* and kanamycin resistance gene from pTnMod-Okm | This study |
| pEX18Tc-*ndpD* | *ndpD* gene knockout vector containing two DNA fragments homologous to the upstream and downstream regions of the *ndpD* and kanamycin resistance gene from pTnMod-Okm | This study |
| pRK415 | Broad host range vector , Tc^r^ | (Keen et al. 1988) |
| pRK415-*ndpA_L_* | *ndpA_L_* gene complementation vector by fusing *ndpA_L_* into the *Hin*d III-*EcoR* I restriction site of pRK415 | This study |
| pRK415-*ndpA* | Heterologous expression vector with *ndpA* insert into the *Hin*d III-*Eco*R I restriction site of pRK415 |  |
| pRK415-*npdA_plus_* | Heterologous expression vector by adding an extended segment of 223 bp in 5’ of *ndpA* compared with pRK415-*ndpA* |  |
| pRK415-*ndpB* | *ndpB* gene complementation vector by fusing *ndpB* into the *Hin*d III-*EcoR* I restriction site of pRK415 | This study |
| pRK415-*ndpB_his_* | *ndpB* gene with a 6-Histag insert into the *Hin*d III-*EcoR* I restriction site of pRK415 | This study |
| pRK415-*ndpC* | *ndpC* gene complementation vector by fusing *ndpC* into the *Hin*d III-*EcoR* I restriction site of pRK415 | This study |
| pRK415-*ndpD* | *ndpD* gene complementation vector by fusing *ndpD* into the *Hin*d III-*EcoR* I restriction site of pRK415 | This study |
| pET-28a(+) | Expression vector, Kan^r^, C/N-terminal His•Tag/thrombin/T7•Tag, T7 *lac* promoter, T7 transcription start, f1 origin, *lacI* | Novagen |
| pET28a-*ndpB* | Expression vector for *ndpB* with C-terminal His•Tag by cloning *ndpB* into the *Nco* I-*Hin*d III restriction site | This study |
| pET28a-*ndpD*-C | Expression vector for *ndpD* with C-terminal His•Tag by cloning *ndpD* into the *Nco* I-*Hin*d III restriction site | This study |
| pET28a-*ndpD*-N | Expression vector for *ndpD* with N-terminal His•Tag by cloning *ndpD* into the *Nde* I- *Xho* I restriction site | This study |
| pET-22b(+) | Expression vector, Amp^r^, C-terminal His•Tag, T7 *lac* promoter, *pelB* signal sequence | Novagen |
| pET22b-*ndpB* | Expression vector for *ndpB* with C-terminal His•Tag by cloning *ndpB* into the *Nco* I-*Hin*d III restriction site | This study |

Dehio C, Meyer M (1997) Maintenance of broad-host-range incompatibility group P and group Q plasmids and transposition of Tn5 in Bartonella henselae following conjugal plasmid transfer from Escherichia coli. J Bacteriol 179(2 %U <http://jb.asm.org/content/179/2/538.abstract):538-40>

Dennis JJ, Zylstra GJ (1998) Plasposons: Modular self-cloning minitransposon derivatives for rapid genetic analysis of gram-negative bacterial genomes. Appl Environ Microbiol 64(7 %U http://aem.asm.org/content/64/7/2710.abstract):2710-2715

Hoang TT, Karkhoff-Schweizer RR, Kutchma AJ, Schweizer HP (1998) A broad-host-range Flp-FRT recombination system for site-specific excision of chromosomally-located DNA sequences: application for isolation of unmarked *Pseudomonas aeruginosa* mutants. Gene 212(1):77-86 doi:http://dx.doi.org/10.1016/S0378-1119(98)00130-9

Keen NT, Tamaki S, Kobayashi D, Trollinger D (1988) Improved broad-host-range plasmids for DNA cloning in Gram-negative bacteria. Gene 70(1):191-197 doi:http://dx.doi.org/10.1016/0378-1119(88)90117-5

Lee J-S, Shin YK, Yoon J-H, Takeuchi M, Pyun Y-R, Park Y-H (2001) *Sphingomonas aquatilis* sp. nov., *Sphingomonas koreensis* sp. nov., and *Sphingomonas taejonensis* sp. nov., yellow-pigmented bacteria isolated from natural mineral water. Int J Syst Evol Microbiol 51(4):1491-1498

Nelson K, Weinel C, Paulsen I, Dodson R, Hilbert H, Martins dos Santos V, Fouts D, Gill S, Pop M, Holmes M (2002) Complete genome sequence and comparative analysis of the metabolically versatile *Pseudomonas putida KT2440*. Environ Microbiol 4(12):799-808

Saltikov CW, Newman DK (2003) Genetic identification of a respiratory arsenate reductase. Proc Natl Acad Sci U S A 100(19):10983-10988 doi:10.1073/pnas.1834303100

Woodcock DM, Crowther PJ, Doherty J, Jefferson S, DeCruz E, Noyer-Weidner M, Smith SS, Michael MZ, Graham MW (1989) Quantitative evaluation of Escherichia coli host strains for tolerance to cytosine methylation in plasmid and phage recombinants. Nucleic Acids Res 17(9 %U <http://nar.oxfordjournals.org/content/17/9/3469.abstract):3469-3478>
