# Supplementary material for: Characterization of a Novel Nicotine Degradation Gene Cluster ndp in Sphingomonas melonis TY and Its Evolutionary Analysis
Source: Front Microbiol. 2017 Mar 9;8:337. doi: 10.3389/fmicb.2017.00337 (PMC5343071; doi:10.3389/fmicb.2017.00337)
Supplement: Supplementary file 2 [file Table2.DOCX]

**Table S2** Oligonucleotide primers used in this study

| Primer name | Primer sequence (5’-3’) | Purpose |
| --- | --- | --- |
| Gene knockout and complementation | | |
| Kan01 | tgtctcaaaatctctgatgttac | To amplify kanamycin resistance gene from pTnMod-Okm for gene knockout |
| Kan02 | ttagaaaaactcatcgagcatc |  |
| ndpA_L_upF^a^ | atgattacgaattcgggcaatcagcctgtgcggttcg | To amplify upstream fragment of *ndpA_L_* for gene knockout |
| ndpA_L_upR | agagattttgagacaagcggccatcgacttcagaagg |  |
| ndpA_L_downF | gatgagtttttctaaagccggagatgtcgagcgcccg | To amplify downstream fragment of *ndpA_L_* for gene knockout |
| ndpA_L_downR | ggccagtgccaagctgtgctgcatgccctgcagatgc |  |
| ndpBupF | atgattacgaattcgcggcattctcaatcccta | To amplify upstream fragment of *ndpB* for gene knockout |
| ndpBupR | gatgagtttttctaaccgctcgcccagcccccgcg |  |
| ndpBdownF | agagattttgagacactcctgcctcccttccgaacct | To amplify downstream fragment of *ndpB* for gene knockout |
| ndpBdownR | ggccagtgccaagctatcagaggtatcaggttctacgc |  |
| ndpCupF | atgattacgaattcgcatcccggcatggtgcat | To amplify upstream fragment of *ndpC* for gene knockout |
| ndpCupR | atgattacgaattcgcatcccggcatggtgcat |  |
| ndpCdownF | agagattttgagacagatgtcaaacccggcccgttt | To amplify downstream fragment of *ndpC* for gene knockout |
| ndpCdownR | ggccagtgccaagctatgccgcgtgttcgcaaatg |  |
| ndpDupF | atgattacgaattcggagccgatcacctacacgat | To amplify upstream fragment of *ndpD* for gene knockout |
| ndpDupR | agagattttgagacatccctctccgtcctggtcga |  |
| ndpDdownF | gatgagtttttctaagcggtgaaccgtcctacgac | To amplify downstream fragment of *ndpD* for gene knockout |
| ndpDdownR | ggccagtgccaagctactttctcgggcttctcctg |  |
| pEX18Tc-VF | gcacgacaggtttcccgactg | For the verification of the pEX18Tc-related constructions by PCR or sequencing |
| pEX18Tc-VR | ccgcttctgcgttctgattta |  |
| ndpA_L_-VF | ctgccgaaggtcatctccc | For the verification of strain TYΔ*ndpA_L_* by PCR or sequencing |
| ndpA_L_-VR | ctctggctggaacttggagg |  |
| ndpB-VF | ttcacgttatagtcaagcac | For the verification of strain TYΔ*ndpB* by PCR or sequencing |
| ndpB-VR | ctaggacaggatcagtcatg |  |
| ndpC-VF | atcgtggtgagccactcgat | For the verification of strain TYΔ*ndpC* by PCR or sequencing |
| ndpC-VR | agcgatactccgcttcgatc |  |
| ndpD-VF | tacgcgtgcaccgaccaacc | For the verification of strain TYΔ*ndpD* by PCR or sequencing |
| ndpD-VR | gacctcgtcgaacagctgct |  |
| ndpA_L_-CF | tgattacgccaagcttcgtgtcggccggcgtggtgttcc | To amplify *ndpA_L_* for gene complementation |
| ndpA_L_-CR | gacggccagtgaattctcaggtgaacgagaggtcgtgg |  |
| ndpB-CF | tgattacgccaagcttcatgggcagcatgacgatgagtg | To amplify *ndpB* for gene complementation |
| ndpB-CR | gacggccagtgaattctcatccattgctgctcttctcc |  |
| ndpC-CF | tgattacgccaagcttcttgtccatgcgcgatccccg | To amplify *ndpC* for gene complementation |
| ndpC-CR | gacggccagtgaattctcactcgacggacgtcccgt |  |
| ndpD-CF | tgattacgccaagcttcatgatggccaagcatgtgatcg | To amplify *ndpD* for gene complementation |
| ndpD-CR | gacggccagtgaattctcagaagtgcgtctccatctg |  |
| ndpA-F | tgattacgccaagcttcgtgaatgtcgacgtcgacg | To amplify *ndpA* for heterologously expression |
| ndpA-R | gacggccagtgaattcctgcggacgtctacgcgat |  |
| ndpA_plus_-F | tgattacgccaagcttttggatcggcgacgacgta | To amplify *ndpA_plus_* for heterologously expression |
| ndpA_plus_-R | gacggccagtgaattcctgcggacgtctacgcgat |  |
| pRK415-VF | gcccaatacgcaaaccgcct | For the verification of the pRK415 related plasmids by PCR or sequencing |
| pRK415-VR | gctctcctgttccgaccctg |  |
| Gene expression | | |
| 28ndpB-EF | aggagatataccatgggcagcatgacgatgagtgag | To amplify *ndpB* for expression with pET-28a(+) |
| 28ndpB-ER | gtgcggccgcaagctttccattgctgctcttctcctt |  |
| 28ndpD-EF-C | aggagatataccatgatggccaagcatgtgatc | To amplify *ndpD* for expression with pET-28a(+) |
| 28ndpD-ER-C | gtgcggccgcaagcttgaagtgcgtctccatctgct |  |
| 28ndpD-EF-N | cgcgcggcagccatatggccaagcatgtgatcgtc | To amplify *ndpD* for expression with pET-28a(+) |
| 28ndpD-ER-N | ggtggtggtgctcgatcagaagtgcgtctccatct |  |
| pET28a-VF | ccatacccacgccgaaacaa | For the verification of the pET-28a(+) related  constructions by PCR or sequencing |
| pET28a-VR | aagggaagaaagcgaaaggagc |  |
| 22ndpB-EF | ccggcgatggccatgggcagcatgacgatgagtga | To amplify *ndpB* for expression with pET-22b(+) |
| 22ndpB-EF | gtgcggccgcaagctttccattgctgctcttctcct |  |
| pET22b-VF | ccatacccacgccgaaacaa | For the verification of the pET-22b(+) related  constructions by PCR or sequencing |
| pET22b-VR | aagggaagaaagcgaaaggagc |  |
| ndpB-R | gacggccagtgaattctcagtggtggtggtggtggt  gctcgagtgcggccgcaagctttccattgctgctctt  ctccttc | Paired with ndpCF to amplify *ndpB* for hetereologous expression |
|  |  |  |
|  |  |  |
| RT-qPCR | | |
| RT-ndpA_L_F | actactcgtacccgttta | To amplify 167 bp fragment in *ndpA_L_* for RT-qPCR |
| RT-ndpA_L_R | gttgaactggaccttctc |  |
| RT-ndpA_S_F | ctcgacatcaccacaatc | To amplify 147 bp fragment in *ndpA_S_* for RT-qPCR |
| RT-ndpA_S_R | gaatgccttcttggacag |  |
| RT-ndpBF | catcaagcagaacatcgg | To amplify 151 bp fragment in *ndpB* for RT-qPCR |
| RT-ndpBR | gtcctggtcgttaatgtc |  |
| RT-ndpCF | gtagtcaactcgatgaac | To amplify 111 bp fragment in *ndpC* for RT-qPCR |
| RT-ndpCR | gttctctcttccatccag |  |
| RT-ndpHF | atcatgtcgatgggcaat | To amplify 175 bp fragment in *ndpH* for RT-qPCR |
| RT-ndpHR | tcagcggcttcatgtaag |  |
| RT-ndpFF | ggtggatacgggtacaac | To amplify 219 bp fragment in *ndpF* for RT-qPCR |
| RT-ndpFR | gagcgagtagtcgagatc |  |
| RT-ndpEF | catcttcctgctcttctc | To amplify 174 bp fragment in *ndpE* for RT-qPCR |
| RT-ndpER | gtgatgtgcatcgttctc |  |
| RT-ndpGF | ttcactgacactcgctac | To amplify 185 bp fragment in *ndpG* for RT-qPCR |
| RT-ndpGR | acaaggagtgccttcatg |  |
| RT-ndpDF | catgctggaattctacga | To amplify 187 bp fragment in *ndpD* for RT-qPCR |
| RT-ndpDR | cttggtgaagctgagttc |  |

^a^: 15bp overlap (5’) in the sequences of the primers for in-fusion cloning is underlined.
