# Supplementary material for: Characterization of a Novel Nicotine Degradation Gene Cluster ndp in Sphingomonas melonis TY and Its Evolutionary Analysis
Source: Front Microbiol. 2017 Mar 9;8:337. doi: 10.3389/fmicb.2017.00337 (PMC5343071; doi:10.3389/fmicb.2017.00337)
Supplement: Supplementary file 3 [file Table3.DOCX]

**Table S3** Gram staining properties of nicotine-degrading strains, strains carrying homologues of *ndpHFEG* and nicotinic acid-degrading strains via the 2, 5-DHP pathway

| Strain | Pathway | Gram staining | Reference |
| --- | --- | --- | --- |
| Nicotine-degrading strains |  |  |  |
| *Pseudomonas* sp. No.41 | PRL^a^ | G- | (1, 2) |
| *Pseudomonas convexa* Pc1 | PRL | G- | (3, 4) |
| *Pseudomonas putida* NRRL B-8061 | PRL | G- | (5) |
| *Pseudomonas* sp. S16 | PRL | G- | (6) |
| *Pseudomonas* sp. strain HF-1 | PRL | G- | (7) |
| *Pseudomonas* sp. HZN6 | PRL | G- | (8) |
| *Agrobacterium* sp. strain S33 | VPP^b^ | G- | (9) |
| *Sphingomonas* sp. TY | VPP | G- | (10) |
| *Shinella* sp. HZN7 | VPP | G- | (11) |
| *Ochrobactrum* sp. Strain SJY1 | VPP | G- | (12) |
| *Arthrobacter oxydans* P-34 | PD^c^ | G+ | (13) |
| *Arthrobacter oxidans* pAO1 | PD | G+ | (14) |
| *Nocardioides* sp. JS614 | PD | G+ | (15) |
| *Rhodococcus opacus* B4 | PD | G+ | (16) |
| *Arthrobacter aurescens* M2012083 | PD | G+ | (17, 18) |
|  |  |  |  |
| Strains with homologous of *ndpHFEG* |  |  |  |
| *Ralstonia pickettii* DTP0602 |  | G- |  |
| *Burkholderia cenocepacia* J2315 |  | G- |  |
| *Burkholderia cenocepacia* MC0 3 |  | G- |  |
| *Burkholderia cenocepacia* HI2424 |  | G- |  |
| *Ralstonia eutropha* H16 |  | G- |  |
| *Burkholderia cenocepacia* AU 1054 |  | G- |  |
| *Ralstonia eutropha* JMP134 |  | G- |  |
| *Streptomyces rapamycinicus* NRRL 5491 |  | G- |  |
|  |  |  |  |
| Nicotinic acid-degrading strains with the 2, 5-DHP formation |  |  |  |
| *Pseudomonas putida KT2440* and other 12 strains with the *nic* cluster^d^ |  | G- | (19) |
| *Pseudomonas fluorescens* |  | G- | (20, 21) |
| *Rhizobium* sp. Strain ORS571 |  | G- | (22) |
| *Pseudomonas fluorescens* TN5 |  | G- | (23) |
| *Pseudomonas convexa* |  | G- | (24) |
| *Pseudomonas putida* N-9 |  | G- | (25) |
| *Pseudomonas fluorescens* KB1 |  | G- | (26) |
| *Sinorhizobium* sp. L1 |  | G- | (27) |

^a^, pyrrolidine pathway; ^b^, VPP pathway; ^c^, pyridine pathway; ^d^, these 12 strains were *Bordetella pertussis* Tohama I, *Bordetella parapertussis* 12822, *Bordetella bronchiseptica* RB50, *Burkholderia cenocepacia* PC184, *Ralstonia eutropha* JMP134, *Comamonas testosteroni* KF-1, *Acidovorax avenae* sbsp. citrulli AAC00–1, *Delftia acidovorans* SPH-1, *Burkholderia xenovorans* LB400, *Pseudomonas putida* GB-1, *Pseudomonas putida* W619 and *Pseudomonas putida* F1, respectively.

**References**

1. **Wada E, Yamasaki K.** 1953. Mechanism of Microbial Degradation of Nicotine. Science **117:**152-153.

2. **Wada E, Yamasaki K.** 1954. Degradation of nicotine by soil bacteria1. J Am Chem Soc **76:**155-157.

3. **Thacker R, Rørvig O, Kahlon P, Gunsalus IC.** 1978. NIC, a conjugative nicotine-nicotinate degradative plasmid in *Pseudomonas convexa*. J Bacteriol **135:**289-290.

4. **Thacker R.** 1969. Conversion of L-Hydroxyproline to glutamate by extracts of strains of *Pseudomonas convexa* and *Pseudomonas fluorescens*. Arch Mikrobiol **64:**235-238.

5. **DeTraglia MC, Tometsko AM.** 1980. Separation of d-(+)-Nicotine from a Racemic Mixture by Stereospecific Degradation of the l-(−) Isomer with *Pseudomonas putida*. Appl Environ Microbiol **39:**1067-1069.

6. **Wang SN, Xu P, Tang HZ, Meng J, Liu XL, Huang J, Chen H, Du Y, Blankespoor HD.** 2004. Biodegradation and detoxification of nicotine in tobacco solid waste by a *Pseudomonas* sp. Biotechnol Lett **26:**1493-1496.

7. **Ruan A, Min H, Peng X, Huang Z.** 2005. Isolation and characterization of *Pseudomonas* sp. strain HF-1, capable of degrading nicotine. Res Microbiol **156:**700-706.

8. **Qiu J, Ma Y, Chen L, Wu L, Wen Y, Liu W.** 2011. A sirA-like gene, sirA2, is essential for 3-succinoyl-pyridine metabolism in the newly isolated nicotine-degrading *Pseudomonas* sp. HZN6 strain. Appl Microbiol Biotechnol **92:**1023-1032.

9. **Wang SN, Liu Z, Xu P.** 2009. Biodegradation of nicotine by a newly isolated *Agrobacterium* sp. strain S33. J Appl Microbiol **107:**838-847.

10. **Wang M, Yang G, Wang X, Yao Y, Min H, Lu Z.** 2011. Nicotine degradation by two novel bacterial isolates of *Acinetobacter* sp. TW and *Sphingomonas* sp. TY and their responses in the presence of neonicotinoid insecticides. World J Microbiol Biotechnol **27:**1633-1640.

11. **Ma Y, Wei Y, Qiu J, Wen R, Hong J, Liu W.** 2013. Isolation, transposon mutagenesis, and characterization of the novel nicotine-degrading strain *Shinella* sp. HZN7. Appl Microbiol Biotechnol **98:**2625-2636.

12. **Yu H, Tang H, Zhu X, Li Y, Xu P.** 2014. Molecular mechanism of nicotine degradation by a newly isolated strain *Ochrobactrum* sp. SJY1. Appl Environ Microbiol.

13. **Hochstein LI, Rittenberg SC.** 1959. The bacterial oxidation of nicotine: I. nicotine oxidation by cell-free preparations. J Biol Chem **234:**151-155.

14. **Eberwein H, Gries FA, Decker K.** 1961. Über den Abbau des Nicotins durch Bakterienenzyme, II. Isolierung und Charakterisierung eines nicotinabbauenden Bodenbakteriums. Hoppe-Seyler´ s Zeitschrift für physiologische Chemie **323:**236-248.

15. **Ganas P, Sachelaru P, Mihasan M, Igloi G, Brandsch R.** 2008. Two closely related pathways of nicotine catabolism in *Arthrobacter nicotinovorans* and *Nocardioides* sp. strain JS614. Arch Microbiol **189:**511-517.

16. **Cobzaru C, Ganas P, Mihasan M, Schleberger P, Brandsch R.** 2011. Homologous gene clusters of nicotine catabolism, including a new ω-amidase for α-ketoglutaramate, in species of three genera of Gram-positive bacteria. Res Microbiol **162:**285-291.

17. **Yao Y, Tang H, Ren H, Yu H, Wang L, Xu P.** 2012. Genome sequence of a nicotine-degrading strain of *Arthrobacter*. J Bacteriol **194:**5714-5715.

18. **Yao Y, Tang H, Su F, Xu P.** 2015. Comparative genome analysis reveals the molecular basis of nicotine degradation and survival capacities of *Arthrobacter*. Sci Rep **5**.

19. **Jiménez JI, Canales Á, Jiménez-Barbero J, Ginalski K, Rychlewski L, García JL, Díaz E.** 2008. Deciphering the genetic determinants for aerobic nicotinic acid degradation: the nic cluster from *Pseudomonas putida KT2440*. Proc Natl Acad Sci U S A **105:**11329-11334.

20. **Behrman EJ, Stanier R.** 1957. The bacterial oxidation of nicotinic acid. University of California, Berkeley.

21. **Ensign JC, Rittenberg SC.** 1964. The pathway of nicotinic acid oxidation by a *Bacillus species*. J Biol Chem **239:**2285-2291.

22. **Ludwig RA.** 1986. *Rhizobium* sp. strain ORS571 grows synergistically on N2 and nicotinate as N sources. J Bacteriol **165:**304-307.

23. **Nakano H, Wieser M, Hurh B, Kawai T, Yoshida T, Yamane T, Nagasawa T.** 1999. Purification, characterization and gene cloning of 6‐hydroxynicotinate 3‐monooxygenase from *Pseudomonas fluorescens* TN5. Eur J Biochem **260:**120-126.

24. **Thacker R, Rørvig O, Kahlon P, Gunsalus I.** 1978. NIC, a conjugative nicotine-nicotinate degradative plasmid in *Pseudomonas convexa*. J Bacteriol **135:**289-290.

25. **Gauthier JJ, Rittenberg SC.** 1971. The Metabolism of Nicotinic Acid I. PURIFICATION AND PROPERTIES OF 2, 5-DIHYDROXYPYRIDINE OXYGENASE FROM *PSEUDOMONAS PUTIDA* N-9. J Biol Chem **246:**3737-3742.

26. **Hughes D.** 1955. 6-Hydroxynicotinic acid as an intermediate in the oxidation of nicotinic acid by *Pseudomonas fluorescens*. Biochem J **60:**303.

27. **Karvelis L, Gasparavičiūtė R, Meškys R.** 2012. Characterization of 2, 5-dihydroxypyridine dioxygenases from *Sinorhizobium* sp. L1. Biologija **58**.
