# Supplementary material for: Characterization of a Novel Nicotine Degradation Gene Cluster ndp in Sphingomonas melonis TY and Its Evolutionary Analysis
Source: Front Microbiol. 2017 Mar 9;8:337. doi: 10.3389/fmicb.2017.00337 (PMC5343071; doi:10.3389/fmicb.2017.00337)
Supplement: Supplementary file 4 [file Table4.DOCX]

**Table S4** The basic information for six genes in the nicotine degradation pathway from 50 bacterial genomes

| Kingdom | Phylum | Class | Order | Family | Genus | Species | *ndpA_L_* | *ndpB* | *ndpH* | *ndpF* | *ndpE* | *ndpG* |
| --- | --- | --- | --- | --- | --- | --- | --- | --- | --- | --- | --- | --- |
| Bacteria | Proteobacteria | Gammaproteobacteria | Pseudomonadales | Pseudomonadaceae | *Pseudomonas* | *Pseudomonas_monteilii*_SB3101_uid232253 | NC_023076: X970_19615 | NC_023076: X970_19630 ,X970_19625,X970_23475 | NC_023076: X970_19525 | NC_023076: X970_19520 |  |  |
| Bacteria | Proteobacteria | Gammaproteobacteria | Pseudomonadales | Pseudomonadaceae | *Pseudomonas* | *Pseudomonas_monteilii*_SB3078_uid232252 | NC_023075: X969_19980 | NC_023075: X969_19995, X969_19990, X969_23840 | NC_023075: X969_19890 | NC_023075: X969_19885 |  |  |
| Bacteria | Proteobacteria | Gammaproteobacteria | Pseudomonadales | Pseudomonadaceae | *Pseudomonas* | *Pseudomonas_aeruginosa*_LES431_uid232245 | NC_023066: T223_17600 | NC_023066: T223_02130 |  |  |  | NC_023066: T223_11510 |
| Bacteria | Proteobacteria | Gammaproteobacteria | Pseudomonadales | Pseudomonadaceae | *Pseudomonas* | *Pseudomonas_aeruginosa*_MTB_uid231150 | NC_023019: U769_16255 | NC_023019: U769_02170 |  |  |  |  |
| Bacteria | Proteobacteria | Gammaproteobacteria | Pseudomonadales | Pseudomonadaceae | *Pseudomonas* | *Pseudomonas_aeruginosa*_PA1R_uid228932 | NC_022806: PA1R_gp5507 | NC_022806: PA1R_gp3932 |  |  |  |  |
| Bacteria | Actinobacteria | Actinobacteridae | Actinomycetales | Streptomycetaceae | *Streptomyces* | *Streptomyces_rapamycinicus*_NRRL_5491_uid227224 | NC_022785: M271_44565 | NC_022785: M271_42050 | NC_022785: M271_30770, M271_44540 | NC_022785: M271_11815 ,M271_44545 | NC_022785: M271_44585 | NC_022785: M271_08465, M271_44550 |
| Bacteria | Proteobacteria | Gammaproteobacteria | Pseudomonadales | Pseudomonadaceae | *Pseudomonas* | *Pseudomonas_aeruginosa*_PAO1_VE2_uid225026 | NC_022591: N296_1939 | NC_022591: N296_432 |  |  |  |  |
| Bacteria | Proteobacteria | Betaproteobacteria | Burkholderiales | Burkholderiaceae | *Ralstonia* | *Ralstonia_pickettii*_DTP0602_uid222229 | NC_022515: N234_37005 ;NC_022514: N234_24900 N234_23505 | NC_022515: N234_34600, N234_34540 | NC_022513: N234_03080 ,N234_04320, N234_09685; NC_022514: N234_23140, N234_24880 | NC_022513: N234_04325; NC_022514: N234_24885 | NC_022514: N234_24890 | NC_022513: N234_04335; NC_022515: N234_34900 ;NC_022514: N234_24895 |
| Bacteria | Proteobacteria | Gammaproteobacteria | Pseudomonadales | Pseudomonadaceae | *Pseudomonas* | *Pseudomonas_aeruginosa*_PAO581_uid219357 | NC_022361: M801_1938 | NC_022361: M801_432 |  |  |  |  |
| Bacteria | Proteobacteria | Gammaproteobacteria | Pseudomonadales | Pseudomonadaceae | *Pseudomonas* | *Pseudomonas_aeruginosa*_c7447m_uid219358 | NC_022360: M802_1937 | NC_022360: M802_431 |  |  |  |  |
| Bacteria | Proteobacteria | Gammaproteobacteria | Pseudomonadales | Pseudomonadaceae | *Pseudomonas* | *Pseudomonas_aeruginosa*_RP73_uid209328 | NC_021577: M062_09825 | NC_021577: M062_02115 |  |  |  | NC_021577: M062_14895 |
| Bacteria | Proteobacteria | Gammaproteobacteria | Pseudomonadales | Pseudomonadaceae | *Pseudomonas* | *Pseudomonas_aeruginosa*_B136_33_uid196598 | NC_020912: G655_15720 | NC_020912: G655_02145 |  |  |  |  |
| Bacteria | Proteobacteria | Betaproteobacteria | Rhodocyclales | Rhodocyclaceae | *Azoarcus* | *Azoarcus*_KH32C_uid193704 | NC_020516: AZKH_3902 | NC_020516: AZKH_1882 | NC_020516: AZKH_1740 |  |  | NC_020548: AZKH_p0025 |
| Bacteria | Actinobacteria | Actinobacteridae | Actinomycetales | Mycobacteriaceae | *Mycobacterium* | *Mycobacterium_liflandii*_128FXT_uid59005 | NC_020133: MULP_00619 | NC_020133: MULP_01560 | NC_020133: MULP_03664 |  |  |  |
| Bacteria | Actinobacteria | Actinobacteridae | Actinomycetales | Mycobacteriaceae | *Mycobacterium* | *Mycobacterium_smegmatis*_JS623_uid184820 | NC_019966: Mycsm_05718 | NC_019966: Mycsm_05788 |  | NC_019966: Mycsm_02580 |  | NC_019966: Mycsm_03207 |
| Bacteria | Proteobacteria | Gammaproteobacteria | Pseudomonadales | Pseudomonadaceae | *Pseudomonas* | *Pseudomonas_putida*_UW4_uid182733 | NC_019670: PputUW4_02893 | NC_019670: PputUW4_05086 |  | NC_019670: PputUW4_01605 |  |  |
| Bacteria | Proteobacteria | Betaproteobacteria | Burkholderiales | Burkholderiaceae | *Burkholderia* | *Burkholderia_cepacia*_GG4_uid173858 | NC_018514: GEM_4222 | NC_018514: GEM_5472, GEM_5461 | NC_018514: GEM_5631 |  |  | NC_018514: GEM_3880 |
| Bacteria | Actinobacteria | Actinobacteridae | Actinomycetales | Mycobacteriaceae | *Mycobacterium* | *Mycobacterium_smegmatis*_MC2_155_uid171958 | NC_018289: MSMEI_2401 | NC_018289: MSMEI_1990 | NC_018289: MSMEI_2430 ,MSMEI_2435 ,MSMEI_4492 |  |  | NC_018289: MSMEI_4027 |
| Bacteria | Proteobacteria | Gammaproteobacteria | Pseudomonadales | Pseudomonadaceae | *Pseudomonas* | *Pseudomonas_aeruginosa*_DK2_uid168996 | NC_018080: PADK2_16250 | NC_018080: PADK2_02120 |  |  |  | NC_018080: PADK2_10445 |
| Bacteria | Actinobacteria | Actinobacteridae | Actinomycetales | Mycobacteriaceae | *Mycobacterium* | *Mycobacterium_chubuense*_NBB4_uid168322 | NC_018027: Mycch_4545 | NC_018027: Mycch_4614 ,Mycch_1513 |  |  |  |  |
| Bacteria | Proteobacteria | Alphaproteobacteria | Rhizobiales | Bradyrhizobiaceae | *Bradyrhizobium* | *Bradyrhizobium_japonicum*_USDA_6_uid158851 | NC_017249: BJ6T_70410, BJ6T_33370, BJ6T_21930 | NC_017249: BJ6T_05110 |  | NC_017249: BJ6T_03800, BJ6T_41850, BJ6T_67480 |  | NC_017249: BJ6T_15770, BJ6T_35570, BJ6T_63770 |
| Bacteria | Proteobacteria | Gammaproteobacteria | Pseudomonadales | Pseudomonadaceae | *Pseudomonas* | *Pseudomonas_fluorescens*_F113_uid87037 | NC_016830: PSF113_4159 | NC_016830: PSF113_5508 | NC_016830: PSF113_3457 | NC_016830: PSF113_1874 ,PSF113_2492 |  |  |
| Bacteria | Proteobacteria | Alphaproteobacteria | Rhodobacterales | Rhodobacteraceae | *Pseudovibrio* | *Pseudovibrio*_FO_BEG1_uid82373 | NC_016642: PSE_1025 | NC_016642: PSE_0361 | NC_016642: PSE_1837 |  |  |  |
| Bacteria | Actinobacteria | Actinobacteridae | Actinomycetales | Mycobacteriaceae | *Mycobacterium* | *Mycobacterium_rhodesiae*_NBB3_uid75107 | NC_016604: MycrhN_2358 | NC_016604: MycrhN_0829 ,MycrhN_0284 |  | NC_016604: MycrhN_5581 |  |  |
| Bacteria | Proteobacteria | Gammaproteobacteria | Pseudomonadales | Pseudomonadaceae | *Pseudomonas* | *Pseudomonas_putida*_S16_uid68747 | NC_015733: PPS_4078 | NC_015733: PPS_4081, PPS_4080 ,PPS_4827 | NC_015733: PPS_4060 | NC_015733: PPS_4059 | NC_015733: PPS_4058 | NC_015733: PPS_4057 |
| Bacteria | Proteobacteria | Gammaproteobacteria | Pseudomonadales | Pseudomonadaceae | *Pseudomonas* | *Pseudomonas_brassicacearum*_NFM421_uid66303 | NC_015379: PSEBR_a4062 | NC_015379: PSEBR_a5290 |  | NC_015379: PSEBR_a2349 ,PSEBR_a3895 |  | NC_015379: PSEBR_a2112 |
| Bacteria | Actinobacteria | Actinobacteridae | Actinomycetales | Mycobacteriaceae | *Mycobacterium* | *Mycobacterium_gilvum_Spyr1*_uid61403 | NC_014814: Mspyr1_09570 | NC_014814: Mspyr1_49510 |  | NC_014814: Mspyr1_32970 ,Mspyr1_48290 |  |  |
| Bacteria | Proteobacteria | Alphaproteobacteria | Rhodospirillales | Rhodospirillaceae | *Azospirillum* | *Azospirillum*_B510_uid46085 | NC_013859: AZL_e03260 ;NC_013857: AZL_c02260 | NC_013856: AZL_b03560 |  | NC_013855: AZL_a02710 |  |  |
| Bacteria | Actinobacteria | Actinobacteridae | Actinomycetales | Streptosporangiaceae | *Streptosporangium* | *Streptosporangium_roseum*_DSM_43021_uid42521 | NC_013595: Sros_6667 ,Sros_5751, Sros_1219 | NC_013595: Sros_5603 | NC_013595: Sros_6615 | NC_013595: Sros_1693 |  | NC_013595: Sros_4611 ,Sros_6622 |
| Bacteria | Actinobacteria | Actinobacteridae | Actinomycetales | Nocardiaceae | *Rhodococcus* | *Rhodococcus_opacus*_B4_uid13791 | NC_012522: ROP_27550, ROP_27460 | NC_012522: ROP_32040 ,ROP_33710, ROP_01180 ,ROP_04530, ROP_57750 | NC_012522: ROP_34370, ROP_44900, ROP_44910 | NC_012522: ROP_02980 |  | NC_012522: ROP_71480 |
| Bacteria | Chloroflexi | Chloroflexales | Chloroflexales | Chloroflexaceae | *Chloroflexus* | *Chloroflexus*_Y_400_fl_uid59085 | NC_012032: Chy400_3737, Chy400_2270 | NC_012032: Chy400_1500 |  |  |  | NC_012032: Chy400_2435 |
| Bacteria | Chloroflexi | Chloroflexales | Chloroflexales | Chloroflexaceae | *Chloroflexus* | *Chloroflexus_aggregans*_DSM_9485_uid58621 | NC_011831: Cagg_0973, Cagg_1559 | NC_011831: Cagg_2955 |  |  |  | NC_011831: Cagg_3089 |
| Bacteria | Proteobacteria | Gammaproteobacteria | Pseudomonadales | Pseudomonadaceae | *Pseudomonas* | *Pseudomonas_aeruginosa*_LESB58_uid59275 | NC_011770: PLES_34431 | NC_011770: PLES_04191 |  |  |  |  |
| Bacteria | Proteobacteria | Betaproteobacteria | Burkholderiales | Burkholderiaceae | *Burkholderia* | *Burkholderia_cenocepacia*_J2315_uid57953 | NC_011001: BCAM1540 ;NC_011000: BCAL1075 | NC_011001: BCAM0112, BCAM0123 | NC_011001: BCAM1144 ;NC_011000: BCAL1155 | NC_011002: BCAS0570 ;NC_011001: BCAM1143; NC_011000: BCAL0455 ,BCAL2578 | NC_011001: BCAM1142 | NC_011001: BCAM1141, BCAM1751 |
| Bacteria | Actinobacteria | Actinobacteridae | Actinomycetales | Mycobacteriaceae | *Mycobacterium* | *Mycobacterium_marinum*_M_uid59423 | NC_010612: MMAR_0623 | NC_010612: MMAR_1389 | NC_010612: MMAR_3401 |  |  |  |
| Bacteria | Proteobacteria | Betaproteobacteria | Burkholderiales | Burkholderiaceae | *Burkholderia* | *Burkholderia_cenocepacia*_MC0_3_uid58769 | NC_010515: Bcenmc03_5950 ,Bcenmc03_5083 | NC_010515: Bcenmc03_4367 | NC_010512: Bcenmc03_6238; NC_010515: Bcenmc03_3484 | NC_010512: Bcenmc03_7003 ;NC_010515: Bcenmc03_3485 | NC_010515: Bcenmc03_3486 | NC_010515: Bcenmc03_3487 ,Bcenmc03_4208, Bcenmc03_5339, Bcenmc03_5733 |
| Bacteria | Chloroflexi | Chloroflexales | Chloroflexales | Chloroflexaceae | *Roseiflexus* | *Roseiflexus_castenholzii*_DSM_13941_uid58287 | NC_009767: Rcas_2239, Rcas_0842 | NC_009767: Rcas_2074 |  | NC_009767: Rcas_1483 |  | NC_009767: Rcas_3491 |
| Bacteria | Proteobacteria | Gammaproteobacteria | Pseudomonadales | Pseudomonadaceae | *Pseudomonas* | *Pseudomonas_aeruginosa*_PA7_uid58627 | NC_009656: PSPA7_3406 | NC_009656: PSPA7_0521 |  |  |  |  |
| Bacteria | Actinobacteria | Actinobacteridae | Actinomycetales | Mycobacteriaceae | *Mycobacterium* | *Mycobacterium_gilvum*_PYR_GCK_uid59421 | NC_009338: Mflv_1573 | NC_009338: Mflv_1230 |  | NC_009338: Mflv_1348, Mflv_3952 |  |  |
| Bacteria | Actinobacteria | Actinobacteridae | Actinomycetales | Pseudonocardiaceae | *Saccharopolyspora* | *Saccharopolyspora_erythraea*_NRRL_2338_uid62947 | NC_009142: SACE_4019 ,SACE_4017 | NC_009142: SACE_4150 ,SACE_2785 SACE_6018 | NC_009142: SACE_3770 ,SACE_4875 |  |  | NC_009142: SACE_1113 |
| Bacteria | Actinobacteria | Actinobacteridae | Actinomycetales | Mycobacteriaceae | *Mycobacterium* | *Mycobacterium_vanbaalenii*_PYR_1_uid58463 | NC_008726: Mvan_5183 | NC_008726: Mvan_1178, Mvan_5578 ,Mvan_1866 |  | NC_008726: Mvan_2445 |  |  |
| Bacteria | Actinobacteria | Actinobacteridae | Actinomycetales | Nocardioidaceae | *Nocardioides* | *Nocardioides*_JS614_uid58149 | NC_008699: Noca_0596 ,Noca_1491, Noca_0612 | NC_008699: Noca_0586 |  |  |  |  |
| Bacteria | Actinobacteria | Actinobacteridae | Actinomycetales | Mycobacteriaceae | *Mycobacterium* | *Mycobacterium_ulcerans*_Agy99_uid62939 | NC_008611: MUL_0588 | NC_008611: MUL_2487 |  |  |  |  |
| Bacteria | Actinobacteria | Actinobacteridae | Actinomycetales | Mycobacteriaceae | *Mycobacterium* | *Mycobacterium_smegmatis*_MC2_155_uid57701 | NC_008596: MSMEG_2462 | NC_008596: MSMEG_2035 | NC_008596: MSMEG_2490 ,MSMEG_2495, MSMEG_4610 |  |  | NC_008596: MSMEG_4126 |
| Bacteria | Proteobacteria | Betaproteobacteria | Burkholderiales | Burkholderiaceae | *Burkholderia* | *Burkholderia_cenocepacia*_HI2424_uid58369 | NC_008543: Bcen2424_4407 ,Bcen2424_5194 | NC_008543: Bcen2424_5812 | NC_008544: Bcen2424_6640; NC_008543: Bcen2424_4041 | NC_008544: Bcen2424_6337 ,Bcen2424_6723; NC_008543: Bcen2424_4040 | NC_008543: Bcen2424_4039 | NC_008543: Bcen2424_4038 ,Bcen2424_4572 ,Bcen2424_4947 |
| Bacteria | Proteobacteria | Betaproteobacteria | Burkholderiales | Burkholderiaceae | *Cupriavidus* | *Ralstonia_eutropha*_H16_uid62925 | NC_008314: H16_B0815 | NC_008313: H16_A0856, H16_A0845 | NC_008314: H16_B0811 ;NC_008313: H16_A0923 | NC_008314: H16_B0812; NC_008313: H16_A0924 | NC_008314: H16_B0813,NC_008313: H16_A0925 | NC_008314: H16_B0623, H16_B0814 ;NC_008313: H16_A0926 ,H16_A1544 |
| Bacteria | Proteobacteria | Betaproteobacteria | Burkholderiales | Burkholderiaceae | *Burkholderia* | *Burkholderia_cenocepacia*_AU_1054_uid58371 | NC_008061: Bcen_3960 ,Bcen_3174 | NC_008061: Bcen_5048 | NC_008062: Bcen_6407 ;NC_008061: Bcen_4325 | NC_008062: Bcen_6488; NC_008061: Bcen_4326 ;NC_008060: Bcen_1492 | NC_008061: Bcen_4327 | NC_008061: Bcen_3421 ,Bcen_3796, Bcen_4328 |
| Bacteria | Proteobacteria | Betaproteobacteria | Burkholderiales | Burkholderiaceae | *Burkholderia* | *Burkholderia*_383_uid58073 | NC_007511: Bcep18194_B1602 | NC_007509: Bcep18194_C6833 | NC_007510: Bcep18194_A4901 ;NC_007511: Bcep18194_B3099 |  |  | NC_007511: Bcep18194_B1272 |
| Bacteria | Proteobacteria | Gammaproteobacteria | Pseudomonadales | Pseudomonadaceae | *Pseudomonas* | *Pseudomonas_fluorescens*_Pf0_1_uid57591 | NC_007492: Pfl01_2174, Pfl01_4169 | NC_007492: Pfl01_2742 ,Pfl01_5286 |  |  |  |  |
| Bacteria | Proteobacteria | Betaproteobacteria | Burkholderiales | Burkholderiaceae | *Cupriavidus* | *Ralstonia_eutropha*_JMP134_uid58047 | NC_007347: Reut_A1744; NC_007348: Reut_B3790 | NC_007336: Reut_C6401 | NC_007347: Reut_A2514 ;NC_007348: Reut_B3786, Reut_B5483 | NC_007347: Reut_A2513; NC_007348: Reut_B3787 | NC_007347: Reut_A2512,NC_007348: Reut_B3788 | NC_007347: Reut_A2511; NC_007348: Reut_B3789 ,Reut_B4618 |
|  |  |  |  |  |  |  |  |  |  |  |  |  |
