# Supplementary material for: Characterization of a Novel Nicotine Degradation Gene Cluster ndp in Sphingomonas melonis TY and Its Evolutionary Analysis
Source: Front Microbiol. 2017 Mar 9;8:337. doi: 10.3389/fmicb.2017.00337 (PMC5343071; doi:10.3389/fmicb.2017.00337)
Supplement: Supplementary file 5 [file Presentation1.PDF]

Principal component analysis

Principal component analysis (PCA) (1) was used to analysis the bias of RSCU for the genes in the VPP pathway. To perform PCA, we first normalized the data using the following equation:

$$z = (x - \mu)/\sigma$$

where μ is the mean of each column and σ is the standard deviation of the column. Next, we performed PCA using MatLab (version 2014b, http://cn.mathworks.com/index.html?s_tid=gn_loc_drop). The first 2 principal components of the data were retained to perform a dimensionality reduction, and the RSCU was visualized in a 2-dimensional plane, as shown in Fig. S1 and Fig. S2.

Details about heterologous expression of *ndpA*, *ndpB* and *ndpD*.

pRK415*ndpA* was generated with fragment of *ndpA* just downstream of the promoter of the vector. And pRK415*ndpA*$_{plus}$ were constructed by adding an extended segment of 223 bp in 5' of *ndpA* compared with pRK415-*ndpA*. Plasmids pRK415-*ndpA* and pRK415*ndpA*$_{plus}$ were transferred into *P. putida* KT2440, and pRK415-*ndpA* into *Sphingomonas aquatilis* JSS7^{T} to generate *P. putida* KT-*ndpA*, *P. putida* KT-*ndpA*$_{plus}$ and *Sphingomonas*-*ndpA*, respectively. The expression of NdpA was induced by adding 0.1 mM IPTG. The cells were harvested by centrifugation at 6, 000 × g for 5 min and washed twice with 12 mM PBS, pH 7.4, and the cell pellets were resuspended with ISM to OD600$_{nm}$ reach at 5.0 (resting cells). Then the ability for nicotine transformation of resting cells was determined. Nicotine was added at a final concentration of 0.1 mg/mL in the resting cells with shaking at 200 rpm under 30 ℃. Samples were taken at regular intervals and used for spectrum scanning and high performance liquid chromatography (HPLC) analysis.

The *ndpB* gene was amplified by PCR with the primers listed in Table 2, and then infused into *Nco* I and *Hin*d III digested pET-28a(+) and pET-22b(+) respectively, resulting in pET28a-*ndpB* and pET22b-*ndpB* as C-terminal His$_6$-tagged fusion proteins. The expression vector pET28a-*ndpB* was transformed to *E. coli* BL21(DE3), and pET22b-*ndpB* was transformed to Origami B(DE3). Different concentration of IPTG (vary from 0.01 to 1 mM), temperature (16 ℃, 20 ℃ and 30 ℃) and rotation speed (120 rpm and 200 rpm) of induction, as well as condition of ultrasonication and lysis buffer were attempted to optimize expression of target protein. The growth of expression strains, the induction and overexpression of NdpB, and the preparation of bacterial lysates were carried out according to Instruction Manual of ProBond™ Purification System (Invitrogen, USA), undergoing hybrid conditions purification procedure. The purified protein was analyzed by sodium dodecyl sulfate-polyacrylamide gel electrophoresis (SDS-PAGE) (according to the instruction manual of Mini-PROTEAN® Tetra Cell), and then desalted and concentrated by Ultracel®-10K Amicon® Ultra Centrifugal Filters (Millipore, Ireland).

Plasmids pET28a-*ndpD*-C and pET22b-*ndpD*-N were sequenced and transformed into *E. coli* BL21(DE3). The cells were grown in 37 ℃ to OD600$_{nm}$ reached at approximately 0.5 then IPTG was added at gradient concentration (0, 0.01, 0.02, 0.05, 0.1, 0.2, 0.5 and 1 mM) after ice-water incubation for 30 min, and then incubated at

16 ℃, 100 rpm for 24 h. Cells was collected by centrifugation at 4 ℃, and washed twice by 50 mM Tris-HCl, pH 8.0. Samples of supernatant and precipitate were made for SDS-PAGE after ultrasonication and high-speed centrifugation.

References

1. **Jolliffe I.** 2002. Principal component analysis. Wiley Online Library.
