# Supplementary material for: Characterization of a Novel Nicotine Degradation Gene Cluster ndp in Sphingomonas melonis TY and Its Evolutionary Analysis
Source: Front Microbiol. 2017 Mar 9;8:337. doi: 10.3389/fmicb.2017.00337 (PMC5343071; doi:10.3389/fmicb.2017.00337)
Supplement: Supplementary file 6 [file Image1.PDF]

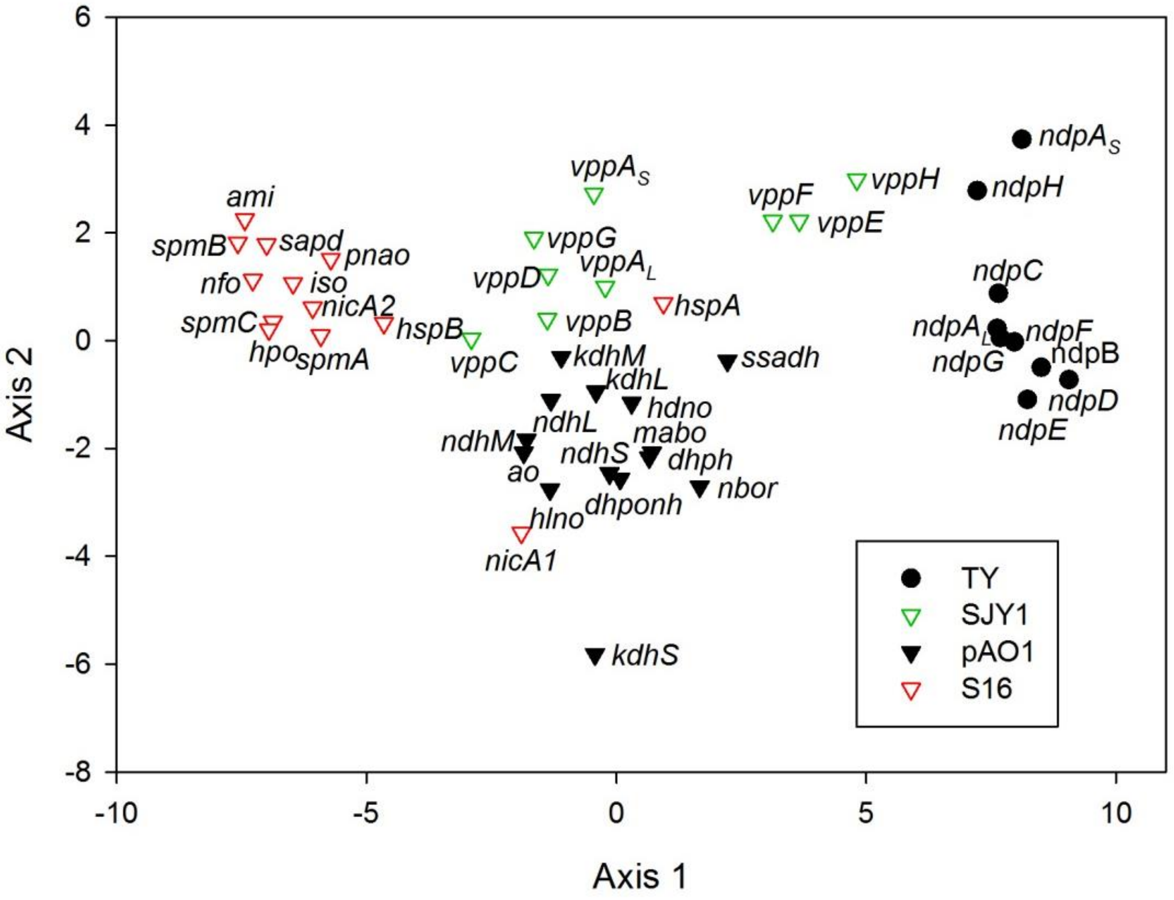

Figure S1 Principal component analysis of the codon usage of nicotine-catalyzing genes in strains TY, SJY1, S16 and *A. nicotinovorans* pAO1. Black dots, genes from strain TY; green triangles, genes from strain SJY1; black triangles, genes from *A. nicotinovorans* pAO1; red triangles, genes from strain S16.
