# Supplementary material for: Characterization of a Novel Nicotine Degradation Gene Cluster ndp in Sphingomonas melonis TY and Its Evolutionary Analysis
Source: Front Microbiol. 2017 Mar 9;8:337. doi: 10.3389/fmicb.2017.00337 (PMC5343071; doi:10.3389/fmicb.2017.00337)
Supplement: Supplementary file 7 [file Image2.PDF]

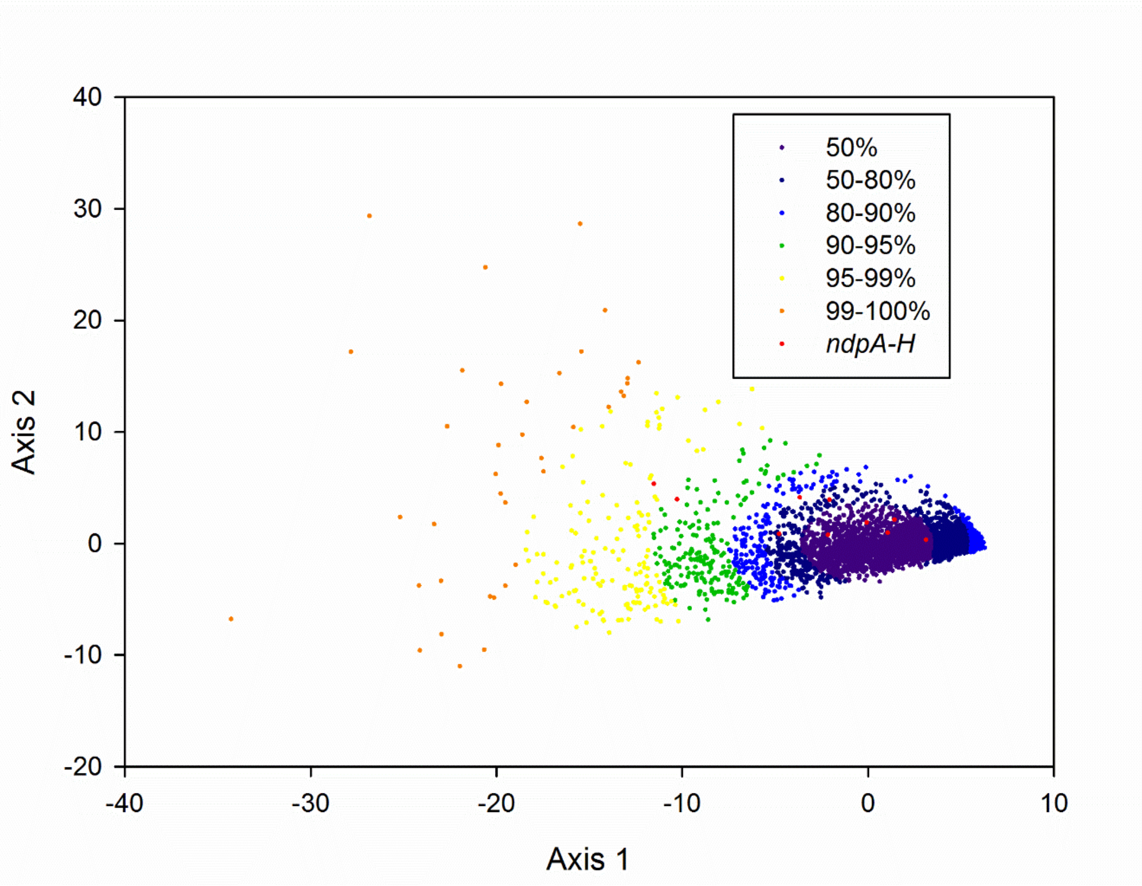

Figure S2 Principal component analysis of the codon usage of 3867 genes in draft genome sequence in strain TY and 10 nicotine-degrading related genes in *ndp*. As shown in the figure, the purple dots mean the 50% of the genes with the relatively similar RSCU; the dark blue dots mean 50-80%; the light blue dots mean 80-90%; the green dots mean 90-95%; the yellow dots mean 95-99%; the orange dots mean 99-100%; the red dots mean 10 nicotine-degrading related genes in *ndp*.
